# *In Vivo* F-Actin Filament Organization during Lymphocyte Transendothelial and Interstitial Migration Revealed by Intravital Microscopy

**DOI:** 10.1016/j.isci.2019.05.040

**Published:** 2019-05-30

**Authors:** Serena L.S. Yan, Il-Young Hwang, Olena Kamenyeva, John H. Kehrl

**Affiliations:** 1 B-cell Molecular Immunology Section, Laboratory of Immunoregulation, National Institutes of Allergy and Infectious Diseases, National Institutes of Health, Bldg. 10, Room 11B08, 10 Center Dr. MSC 1876, Bethesda, MA 20892, USA

**Keywords:** Biological Sciences, Cell Biology, Organizational Aspects of Cell Biology

## Abstract

Actin is essential for many cellular processes including cell motility. Yet the organization of F-actin filaments during lymphocyte transendothelial migration (TEM) and interstitial migration have not been visualized. Here we report a high-resolution confocal intravital imaging technique with LifeAct-GFP bone marrow reconstituted mice, which allowed visualization of lymphocyte F-actin *in vivo*. We find that naive lymphocytes preferentially cross high endothelial venules (HEVs) using paracellular rather than the transcellular route. During both modes of transmigration F-actin levels rise at the lymphocyte leading edge as the cell engages the TEM site. Once the lymphocytes breach the endothelium, they briefly reside in HEV pockets before crossing into the parenchyma. During interstitial migration dynamic actin-based protrusions rapidly form and collapse to help drive motility. Using a panel of inhibitors, we established roles for actin regulators and myosin II in lymphocyte TEM. This study provides further insights into lymphocyte TEM and interstitial migration *in vivo*.

## Highlights

• Established high-resolution imaging technique to visualize HEVs and F-actin *in vivo*
• Naive lymphocytes mainly cross HEVs via paracellular route by breaking junctions
• Rapid re-organization of cellular F-actin during *in vivo* TEM and migration
• *In vivo* F-actin dynamics is important for lymphocyte-endothelium interactions

## Graphical Abstract

**Figure:**
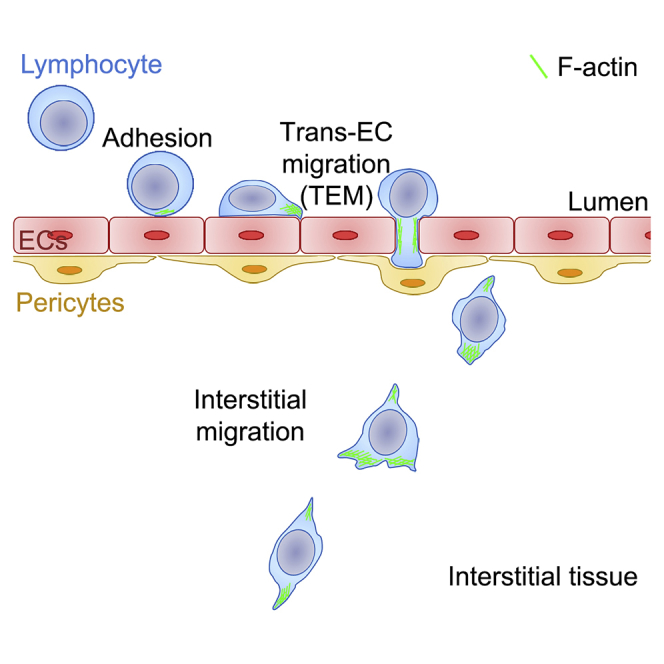

## Introduction

The trafficking of immune cells through lymph nodes (LNs) plays a critical role in immunity. During immune surveillance lymphocytes recirculate from the blood, through LNs, into lymphatics, and back to the blood (Kehrl, 2004, Young, 1999). In a non-inflamed state, millions of naive lymphocytes enter mammalian LNs daily via high endothelial venules (HEVs) and exit via lymphatics (Kehrl, 2006, von Andrian and Mempel, 2003). Lymphocytes access LN parenchyma by migrating through the walls of HEVs in a process called transendothelial migration (TEM) (Girard et al., 2012). TEM occurs through micro-wide gaps in HEVs generated by transmigrating lymphocytes where lymphocytes provide the mechanical force needed to overcome the endothelial cell (EC) barriers allowing their cell body to squeeze through EC gaps and pores (Carman and Springer, 2008, Muller, 2003). Recent studies suggest that the endothelial actin cytoskeletal network maintains the EC cell shape creating a mechanical barrier (Barzilai et al., 2017, Renkawitz and Sixt, 2010). Engagement of transmigrating leukocytes with the endothelium can trigger extensive modifications of EC actin cytoskeleton, including EC contraction and gap openings (Barzilai et al., 2017).

In leukocytes the actin cytoskeleton is involved in cell migration, endocytosis, adhesion, cell synapse formation, and cell division (Billadeau and Burkhardt, 2006, Phng et al., 2013, Vicente-Manzanares and Sanchez-Madrid, 2004). Efficient leukocyte migration is accomplished by a finely regulated cellular cytoskeleton, which allows reorganization of the leukocyte membrane, redistribution of receptors, and cell morphology changes (Mueller et al., 2017, Renkawitz and Sixt, 2010). Leukocyte cytoskeleton-propelled protrusions and deformation are mainly controlled by microfilaments composed of F-actin (Fritz-Laylin et al., 2017, Mueller et al., 2017). Studies have defined many capping, nucleator, and adaptor proteins, which regulate the high rates of actin polymerization and depolymerization that allow the rapid growth and deconstruction of microfilament-based structures (Davidson et al., 2018, Fritz-Laylin et al., 2017, Peng et al., 2011). The cellular actin cytoskeleton arises from the assembly of globular actin (G-actin) into double helical filaments (F-actin) (Huang et al., 2014, Peng et al., 2011). The spontaneous polymerization of G-actin into filaments can be prevented by G-actin-binding and G-actin-sequestering proteins and pharmacological inhibitors (i.e., latrunculin B) (Peng et al., 2011). *De novo* actin polymerization is initiated by actin nucleators or nucleation complexes such as the Arp2/3 and formins (Davidson et al., 2018, Swaney and Li, 2016). Moreover, microfilaments also regulate cell morphology via contraction and relaxation by associating with myosin motor proteins that help generate the mechanical force needed for cell movement (Krummel et al., 2014).

Visualizing the actin cytoskeleton is key for the study of many basic biological processes. The development of LifeAct, a 17-amino-acid peptide, which binds F-actin structures in eukaryotic cells allows for *in vivo* and *in vitro* visualization of actin filament organization (Riedl et al., 2008). To precisely examine the role of lymphocyte actin network and the mechanisms by which lymphocytes transmigrate through HEV ECs, we have established a confocal intravital microscopic imaging system for studying lymphocyte TEM in real time. This imaging technique provides excellent spatial and temporal resolution, and its application to study lymphocyte motility around the HEVs has allowed the accurate analysis of key features of both paracellular and transcellular TEM. Our findings provide direct evidence that proper actin polymerization and function have a key role in supporting lymphocyte transmigration out of the HEVs and suggest that forces generated at the lymphocytes' leading edge by actin cytoskeleton promote the breaching of HEVs either between EC junctions or within an EC body during lymphocyte TEM.

## Results

### Analysis of LifeAct-GFP Expression in Lymphocyte Subsets

**Figure 1:**
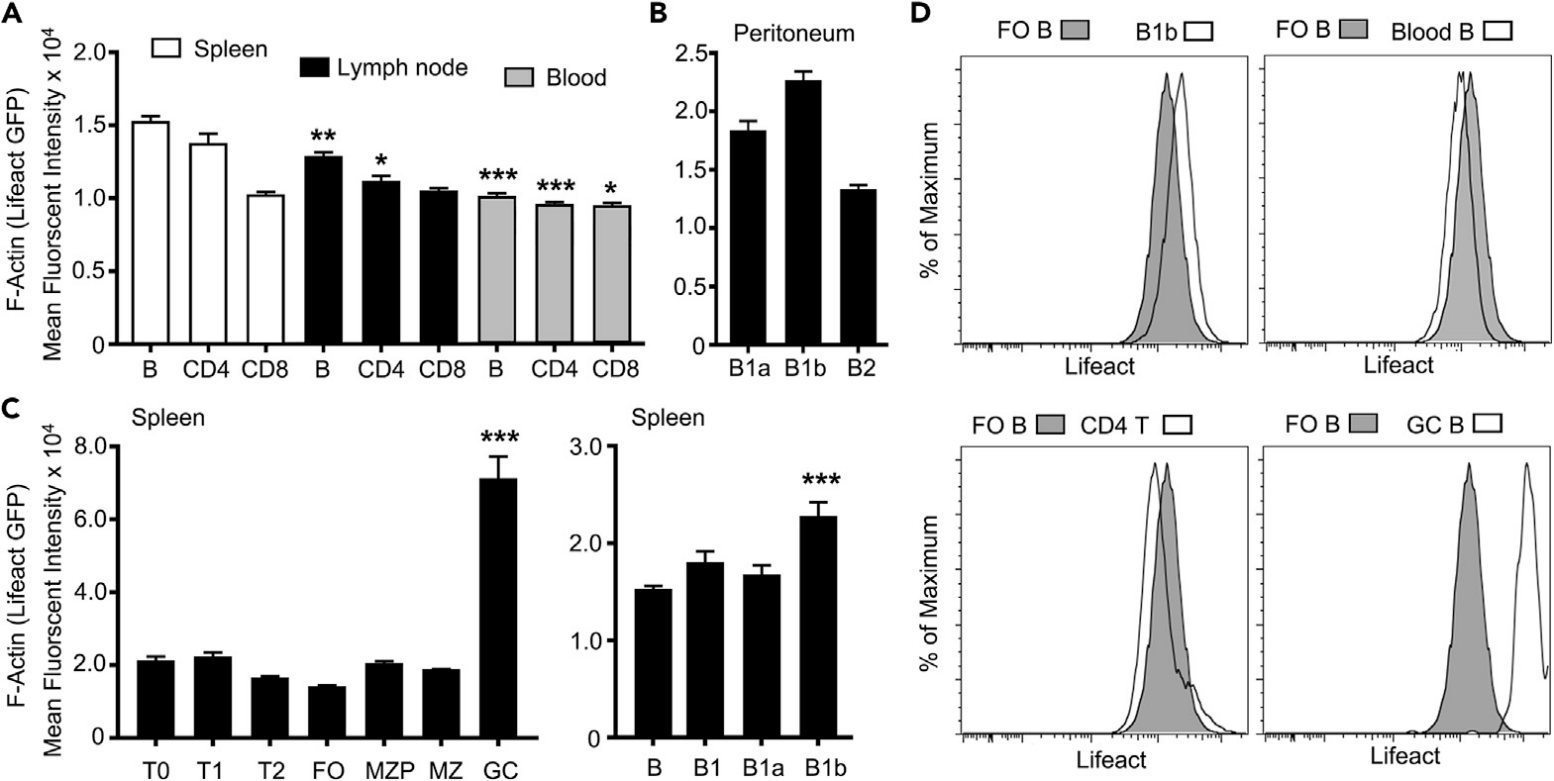
Assessment of LifeAct-GFP Levels in Lymphocyte Subsets (A) Flow cytometry analysis of B, CD4, and CD8 cells' LifeAct-GFP expression levels from spleen, LN, and blood. Statistical analysis comparing cell subsets obtained from LN or blood with the corresponding cell type obtained from spleen. (B) Flow cytometry results showing levels of LifeAct-GFP expression on B1a, B1b, and B2 cells in the peritoneum. (C) Detailed flow cytometric analysis of LifeAct-GFP levels of splenic lymphocyte subsets (T0, T1, T2, FO, MZP, MZ, and GC cells). For the right graph, T1, T2, FO MZP MZ, and GC cells are compared with T0. For the left graph, B1, B2a, and B1b cells are compared with (B). (D) Representative flow cytometry plots showing LifeAct-GFP expression on FO B cells compared with different lymphocyte subsets of interest. Mean fluorescence data are shown as mean ± SEM of the relative expression of LifeAct-GFP (A–C). Negative controls had an average mean fluorescence intensity of 240. Data are representative of three experiments in triplicates. Error bars show mean ± SEM. *p < 0.05, **p < 0.01, ***p < 0.001. Data analysis performed with Prism analysis of variance (ANOVA).

We first assessed the relative expression of LifeAct-GFP in lymphocytes subsets prepared from LifeAct mice using flow cytometry (Riedl et al., 2008). Both blood B and T cells had easily detectable levels of LifeAct-GFP expression as did B and T cells prepared from spleen and LNs (Figure 1A). The overall expression in the splenic lymphocytes slightly exceeded that of the LN cells. B1 cells had more LifeAct expression than conventional B cells in the peritoneum with the levels in B1b exceeding those of B1a cells (Figure 1B). The overall expression levels in the developing B cells in the spleen did not significantly differ. Surprisingly germinal center B cells had much higher levels of LifeAct-GFP compared with the other subsets we analyzed (Figure 1C). Representative flow cytometry plots are shown (Figure 1D). The constitutive LifeAct lymphocyte expression supported the feasibility of *in vivo* imaging studies.

### High-Resolution Confocal Intravital Imaging to Analyze Lymphocyte TEM

**Figure 2:**
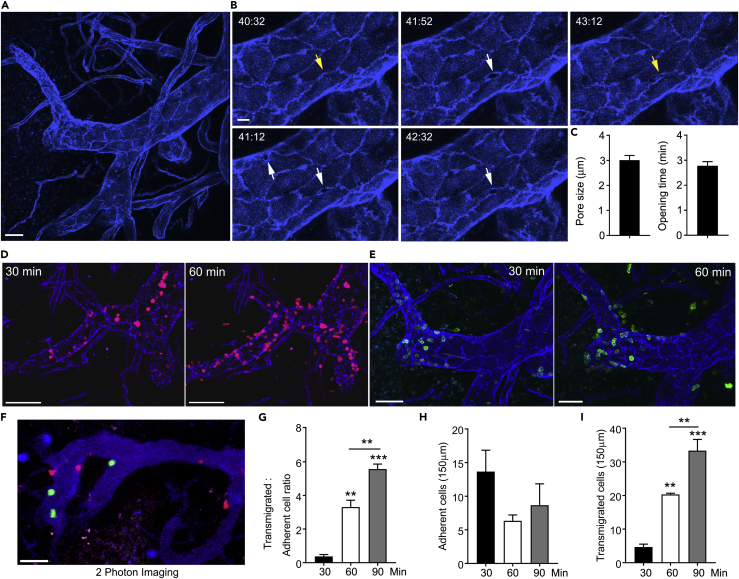
Development of a Four-Dimensional Imaging Platform for the Analysis of Naive Lymphocyte TEM and F-Actin Organization *In Vivo* (A) A representative confocal image of an intravital microscopic experiment of inguinal LN HEVs of wild-type (WT) host mice immunostained *in vivo* for EC junctions by intravenous (i.v.) injection of Alexa Fluor-647-labeled mAb 390 to PECAM-1 (indigo). Scale bar, 15 μm. (B) Representative serial images of confocal intravital microscopy experiments focusing on EC pore openings (min:s). The yellow arrows indicate the location of closed EC junctions that will or was open, and the white arrows indicate the visible pore openings. Scale bar, 6 μm. An overview is shown in Video S1. (C) Quantitative analysis of pore size and pore opening times of 30 events. Error bars show mean ± SEM. (D) Representative images of confocal intravital microscopic experiments of inguinal LN HEVs of WT host mice immunostained with PECAM-1 mAb (CD31, indigo) and CellTracker-labeled lymphocytes (magenta cells). HEVs were imaged at intervals of ∼30 s for a period of ∼60 min with times indicating minutes after i.v. injection of donor lymphocytes. Accumulation and transmigration of lymphocytes in homeostatic HEVs, as shown in Video S2. Scale bar, 60 μm. (E) Representative images of confocal intravital microscopic imaging of WT mice with PECAM-1 immunostaining (indigo) and LifeAct-GFP lymphocyte (green) injection. Times indicate minutes after i.v. injection of LifeAct-GFP donor lymphocytes. Scale bars, 30 μm and 25 μm. (F) Captured intravital two-photon laser scanning microscopy (TP-LSM) image of an inguinal LN HEV with adoptively transferred dye-labeled B cells. The HEVs were outlined by injection of Evans blue (blue). The B cells were adoptively transferred 30 min before imaging. Scale bar, 30 μm. All representative confocal images shown are Z-projections. (G) Ratio comparing total transmigrated with adherent cells over 30, 60, and 90 min after i.v. lymphocyte injection into WT host mice. (H and I) Analysis of adherent lymphocytes that are still firmly attached to the luminal side within the HEVs, and total lymphocyte transmigrated by counting cells outside the HEVs. Adherent (H) and total transmigrated (I) lymphocytes are measured along HEV length of 150 μm as indicated on the y axis. Data are representative of three experiments. Data shown as mean ± SEM, statistics performed with Prism analysis of variance (ANOVA). **p < 0.01, ***p < 0.001.

LN venous blood is collected in one or two large collecting venules (sub-epigastric vein) that drain into the superficial epigastric vein located near the hilus of the LN (Girard et al., 2012, von Andrian, 1996). The HEVs have been ordered by counting successive generations of venular branches in upstream direction from the sub-epigastric vein (order I) (von Andrian, 1996). To study F-actin filament organization during lymphocyte migration through HEVs *in vivo,* we employed a four-dimensional imaging system with advanced spatial and temporal resolution. An essential aspect of the technique was the reproducible and extensive labeling of HEV vasculature and EC junctions (see Transparent Methods). Initially, we intravenously injected a fluorescence-labeled monoclonal antibody (mAb) to platelet endothelial cell adhesion molecule (PECAM)-1 and imaged the inguinal LN HEVs using a multiphoton imaging system (Park et al., 2016, Woodfin et al., 2011). Although we found adequate labeling of the HEV vasculature, the EC contacts in the IV–V ordered HEV venules, the venule segments through which naive lymphocytes predominately transmigrate (Girard et al., 2012, Moratz et al., 2004, von Andrian and Mempel, 2003), were inadequately resolved. Moreover, intravital two-photon imaging of adoptively transferred LifeAct lymphocytes in the inguinal LN poorly visualized the LifeAct signal (data not shown). Therefore we developed an alternative approach. By focusing on HEVs near the cortical medullary junction, 20–40 μm under the LN capsule, we could use a one-photon confocal microscope with highly sensitive detectors. With such an approach, we observed a strong and reliable labeling of the HEV EC borders in the IV – V ordered venules following the intravenous injection of a labeled PECAM-1 mAb (Figure 2A). In addition to observing well-defined EC junctional staining, the labeled ECs showed a faint and dispersed cell body expression of PECAM-1 (Figure S1A). The PECAM-1 antibody labeling of HEV vasculature was specific as a matched control mAb did not outline the vasculature (Figure S1B). The resolving power of the imaging allowed visualization of elliptical pores that transiently opened near EC junctions, consistent with the ongoing transmigration of unlabeled lymphocytes (Figure 2B and Video S1). The long axis of the pores averaged approximately 3 μm, and the pores remained open for 3–4 min (Figure 2C).

Video S1. Visualizing HEV Vasculature and Pore Formation *In Vivo* via PECAM-1 Staining, Related to Figure 2 The video shows the murine inguinal LN vasculature with *in vivo* staining via Alexa Fluor-647-labeled anti-PECAM-1 mAb 390 (white) alone. The video (captured at ∼25X) shows the homeostatic HEV with many movements and activities at the EC level. When zooming in, the transient EC pores are shown with arrows (cyan). Images were captured at 1 frame per 40 s, and the sequence shows a 60-min period. Still images are shown in Figures 2A and 2B.

To provide a control for subsequent experiments with LifeAct lymphocytes, we examined the localization of adoptively transferred, fluorescently labeled lymphocytes relative to PECAM-1-outlined HEVs. At 30 min post transfer, most of the labeled lymphocytes remained confined to the HEVs. By 60 and, more so by 90, min post transfer the lymphocytes had begun to cross the delimited endothelial borders (Figure 2D). Some cells remained confined to the blood vessel wall in HEV pockets, whereas others transmigrated across the pericyte barrier to enter the LN parenchyma (Video S2). Having established that we could image the HEV EC borders in relation to the transferred lymphocytes, we performed similar experiments with adoptively transferred LifeAct lymphocytes. The distribution of the LifeAct lymphocytes mirrored that of the dye-labeled cells. Furthermore, we found that we could assess the distribution of LifeAct-GFP in individual lymphocytes located in HEVs and in those cells that had escaped into the LN parenchyma (Figure 2E). To provide a comparison to past lymphocyte HEV imaging, shown is a representative image obtained during two-photon intravital imaging of fluorescently labeled lymphocytes in the inguinal LN, where the HEVs were outlined by Evans blue dye (Figure 2F).

Video S2. Visualizing Naive Lymphocyte Migration Around an HEV Segment *In Vivo*, Related to Figure 2 The video shows a III & IV-ordered HEV segment within the murine inguinal LN of a WT mouse, immunostained *in vivo* for EC junctions and vascular visualization with Alexa Fluor-647-labeled anti-PECAM-1 mAb 390 (indigo). Splenic lymphocytes isolated from WT donor mouse were labeled with CellTracker Orange (magenta cells) and intravenously injected into the host. The video (captured at ∼25X) shows the homeostatic naive lymphocyte trafficking (starting approximately 30 min post injection of the cells), with lymphocyte adhesion, crawling, transmigration through venular walls, and migration within the interstitial tissue within the LN. Images were captured at 1 frame per 30 s, and the sequence shows a 60-min period. Still images are shown in Figure 2D.

Next, we enumerated the transmigrated and adherent lymphocytes in the HEVs and generated a ratio of transmigrated to adherent cells (Figure 2G). Following adoptive transfer, we observed significant increases in the percentage of transmigrated lymphocytes after 60 and 90 min. Following intravenous injection, adherent lymphocyte rapidly appeared in the HEVs peaking at approximately 30 min post transfer. The number of adherent cells declined as the cells continued to transmigrate across the HEVs (Figure 2H). As expected, the number of transmigrated cells increased as a function of time post transfer (Figure 2I). Having established a robust system for imaging LifeAct lymphocytes in relation to the HEV vasculature, we could examine lymphocyte TEM in greater detail than previously possible.

### Naive Lymphocytes Mainly Exit the HEVs via Paracellular TEM *In Vivo*

**Figure 3:**
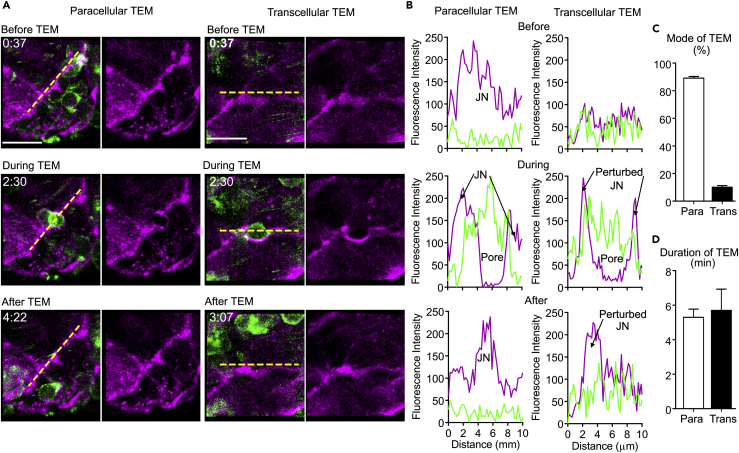
Naive Lymphocyte Paracellular and Transcellular TEM *In Vivo* (A) In-depth analysis of LifeAct-GFP host lymphocytes before, during, and after paracellular (left) and transcellular (right) TEM with indicated times (min:s). Left, transient EC junction pore formation during paracellular TEM (Video S3). Right, transcellular pore is close to EC junctions without disruption of PECAM-1-labeled junctions (Video S4). Dotted yellow lines indicate areas analyzed further in (B). Scale bar, 10 μm. (B) Linear intensity profiles of PECAM-1 channel (EC; magenta) and GFP (lymphocytes, green) of TEM events along the dotted lines in (A); intensity profiles before, during, and after TEM illustrate pore closure in paracellular TEM (left) and shifting of unbroken EC junctions during or after transcellular TEM (right). (C and D) Frequency (C) and duration (D) of TEM events observed and measured in steady-state inguinal LNs. Data are representative of four experiments with >105 TEM events analyzed; error bars (C and D) and SEM calculated with Prism. All representative confocal images shown are Z-projections.

To study the exact route of lymphocyte TEM, i.e., paracellular or transcellular, we created bone marrow chimeric mice using bone marrow from LifeAct-GFP mice. This allowed the visualization of the total host lymphocyte TEM. A limitation of this approach is that all host leukocytes express LifeAct-GFP, but under homeostatic conditions the major leukocyte population transmigrating across HEVs is lymphocytes (Figures S1C and S1D) (Girard et al., 2012). Using these chimeric mice, we assessed the mode of TEM. Most lymphocytes underwent paracellular TEM, where the transient pore formation occurred at the border between two EC (Figure 3A and Video S3). However, a small fraction of lymphocytes crossed the HEVs via transcellular TEM, a non-junctional route. This occurred through a transient pore formation in the EC body, although usually near the EC border (Figure 3A and Video S4). Next, we analyzed the TEM events in greater detail for both modes by visualizing EC pores along with transmigrating LifeAct-GFP lymphocytes (Videos S5 and S6). We generated linear intensity profiles for each transmigration mode (Figure 3B), which revealed paracellular pores closely bordered by junctions with strong PECAM-1 labeling, whereas transcellular pores formed in the EC body close to EC junction with low-intensity PECAM-1 labeling (Woodfin et al., 2011). The junction appears to be perturbed during the transcellular TEM process. After rigorous cell counting and assessment, we determined that approximately 90% of naive lymphocytes exit the HEVs via paracellular TEM, whereas approximately 10% cross via a transcellular route (Figure 3C). Surprisingly, there were no apparent difference in the duration of TEM (4–7 min) between the two routes (Figure 3D). These results indicate that under homeostatic conditions lymphocytes preferentially cross HEVs via the paracellular route through endothelial junctions rich in PECAM-1 expression.

Video S3. Paracellular TEM of Naive Lymphocyte and Junctional Pore Formation, Related to Figure 3 The left panel of this video shows a higher magnification (∼75X) capture of an endogenous naive lymphocyte (green cell) undergoing paracellular TEM in a LifeAct-GFP host mouse immunostained *in vivo* for EC junctions with Alexa Fluor-555-labeled anti-PECAM-1 mAb 390 (magenta). The video taken from the luminal side of the HEV shows an incoming circulating lymphocyte adhering to HEV at EC junctions, breaking the junctions between the two adjacent ECs, and migrating through the EC junctional pore via a paracellular route. The right panel focuses on the pore formation on the HEV during a paracellular TEM event by focusing on visualizing the activity of ECs. By only showing the PECAM-555 channel (magenta) alone, this illustrates the formation of a paracellular pore during lymphocyte TEM. This transient pore formed by breaking EC junctions fused back after the lymphocyte migrated out of the HEV. Images were captured at 1 frame per 60 s and show an ∼7-min period. Representative still images of these sequences are shown in Figure 3A.

Video S4. Transcellular TEM of Naive Lymphocyte and EC Pore Formation via Transcellular TEM, Related to Figure 3 The left panel of this video captures a lymphocyte undergoing transcellular TEM with high magnification (∼75X) in a resting murine inguinal LN from the luminal side of the HEV. The LifeAct-GFP mouse was immunostained *in vivo* for EC junctions with Alexa Fluor-555-labeled anti-PECAM-1 mAb 390 (magenta). The video shows an incoming endogenous circulating lymphocyte adhering to the luminal EC near the junction and then quickly transmigrating out of the HEV by breaching the EC cell body near the junctional border. The right panel focuses on the transient pore formed on the HEV EC during the transcellular TEM event by showing the PECACM-555 channel (magenta) alone. The transient pore formed by visualizing a hole in the EC body and pushing the intact EC junction away during this transcellular TEM event. After the lymphocyte crossed the HEV EC, the pore was resealed with the EC junctions returning to its original pattern. Images were captured at 1 frame per 60 s and show an ∼7-min period. Representative still images of these sequences are shown in Figure 3A.

Video S5. Visualizing Lymphocyte and EC Junction Localization during Paracellular TEM in 3D, Related to Figure 3 This video shows the exact location of lymphocyte wedged between a pore formed by breakage of two adjacent ECs in 3D rotation with high magnification (∼75X). The LifeAct-GFP mouse was immunostained *in vivo* for EC junctions with Alexa Fluor-555-labeled anti-PECAM-1 mAb 390 (magenta). This rotating video illustrates an endogenous LifeAct-GFP lymphocyte (transiently deleted) located within an EC pore formed by breaching EC junctions during a paracellular TEM event. Still images of this event are shown in Figure 3A identified as during TEM, under paracellular TEM.

Video S6. Visualizing Lymphocyte and EC Junction Localization during Transcellular TEM in 3D, Related to Figure 3 This video shows the precise location of lymphocyte caught within a pore formed by breakage of EC body in 3D rotation with high magnification (∼75X). The LifeAct-GFP mouse was immunostained *in vivo* for EC junctions with Alexa Fluor-555-labeled anti-PECAM-1 mAb 390 (magenta). This rotating video illustrates an endogenous LifeAct-GFP lymphocyte (transiently deleted) located within a pore formed on the EC body during a transcellular TEM event, whereas the unbroken EC junction surrounds the transmigrating lymphocyte. Still images of this event are shown in Figure 3B identified as during TEM, under transcellular TEM.

### Visualizing Actin Polymerization *In Vivo* during Lymphocyte TEM

**Figure 4:**
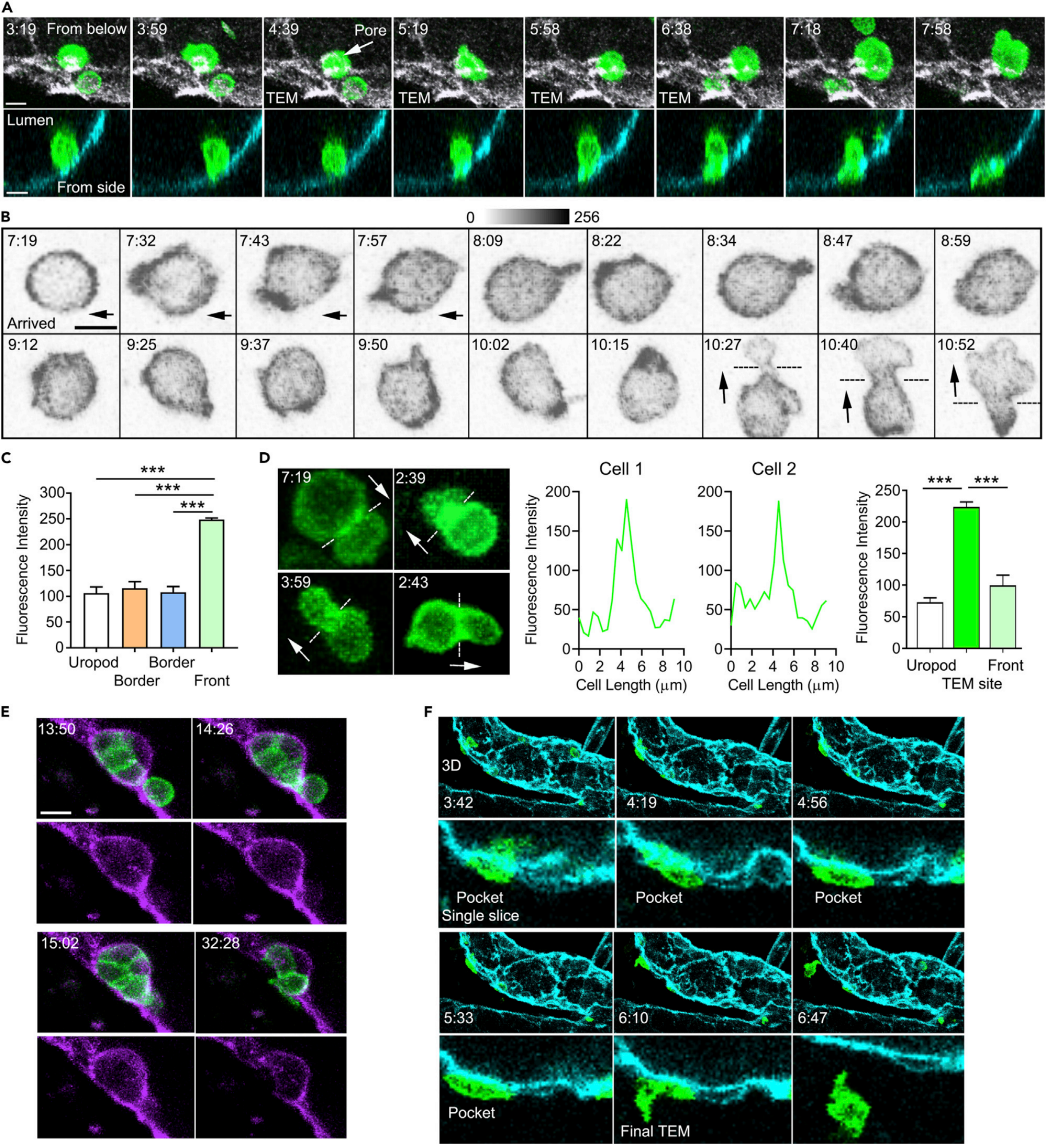
*In Vivo* Imaging of F-Actin Filament Organization during Lymphocyte TEM (A) Time-lapse confocal images of an adoptively transferred LifeAct-GFP lymphocyte undergoing TEM with indicated times (min:s): top, luminal view from below showing PECAM-1 staining (white); bottom, transverse section of venule showing PECAM-1 staining (cyan). Scale bar, 6 μm. (B) Representative time-lapse images of a LifeAct-GFP lymphocyte that found an adjacent TEM site (GFP channel only shown in gray scale with indicated times [min:s]). Black dashed lines indicate the HEV-EC interface. Images collected using Ortho Slicer function in Imaris (4.8 μm/5 slices at 0.96 μm). Scale bar, 6 μm. (C) LifeAct-GFP intensity at the uropod, leading edge, and at the edges perpendicular to the polarity axis as a lymphocyte approaches TEM site. Data are from 30 cells observed and are presented as mean ± SEM. Statistical analysis using Prism (each group compared with the Front via unpaired t test, ***p < 0.001). (D) Representative images of different LifeAct-GFP lymphocytes undergoing TEM (GFP channel only) with indicated times (min:s). White dashed lines indicate the HEV-EC interface, and white arrows show the direction of cell TEM. Images collected using Ortho Slicer function in Imaris (4.8 μm/5 slices at 0.96 μm). Representative LifeAct fluorescence intensity profiles shown for two cells undergoing TEM. Results of analysis of 10 cells (profiles shown in Figure S3). Data shown as mean ± SEM calculated with Prism data analyzed by one-way ANOVA and Tukey's multiple comparison, ***p < 0.001. (E) Time-lapse images of HEV pocket dynamics as LifeAct-GFP lymphocytes (green) accumulate outside the HEV (violet) after TEM with indicated times (min:s), relative to Video S7. Scale bar, 6 μm. (F) Time-lapse images following a LifeAct-GFP lymphocyte (green) undergoing complete TEM from exiting the HEV (cyan) to migrating within the LN interstitium (indicated times, min:s). Top row shows 3D reconstruction, whereas bottom row reveals correlating transverse sections through the middle of the cell. For the early time points see Figure S2. Data are representative of five experiments (A, B, E, and F) with >30 events observed for each phenomenon. All representative confocal images shown are Z-projections.

To examine the behavior of the actin cytoskeleton *in vivo* while lymphocytes undergo TEM, we returned to the adoptively transfer model, transferring LifeAct-GFP lymphocytes into mice with PECAM-1-stained HEVs. Before transmigration, lymphocytes must first firmly adhere to the HEV endothelium (Girard et al., 2012, von Andrian and Mempel, 2003). We found newly adherent lymphocytes non-polarized with a uniform cortical LifeAct-GFP signal. Occasionally newly, firmly adherent cells immediately found a TEM site. The LifeAct-GFP signal quickly intensified in the cell at the site engaged with the endothelium (Figure 4A). Other cells that did not adhere adjacent to a TEM site migrated along the endothelium in search of one (Figure 4B). These cells flattened, polarized, and migrated much like a lymphocyte in a 2D environment, extending a lamellipodia in the direction the cell's migration. The cell shown was imaged directly on the endothelium. After arriving the cell moved to the left, after which it remained relatively stationary. Despite not moving, the cell's most prominent LifeAct signal shifted location until its leading edge engaged the TEM site (Figure 4B, 10:15 time point). The analysis of a typical single lymphocyte as it approaches a TEM site revealed a greater than 2-fold increase in the LifeAct-GFP intensity at the leading edge compared with the uropod (Figures 4C and S2). Once engaged, the cell extended a thin lamellipodia that breached the endothelium. The transmigrating cell propelled itself through the endothelial opening, adopting an hour-glass morphology as it narrowed its cell body to pass through the opening before re-expanding as it emerged into the space between the endothelium and the basement membrane (Figures 4B and 4D). LifeAct-GFP often accumulated in the middle of the cell as it crossed the endothelium before shifting back toward the leading edge or occasionally to the uropod as the cell emerged through the endothelial cell opening (Figure 4D). Once through the endothelium the lymphocytes underwent a second and sometime third transmigration to escape the HEV. Typically, the cell must cross the endothelial cell basement and the pericyte barrier. Some cells enter endothelial pockets where they remain for many minutes. These pockets often contained multiple cells that exhibited dynamic changes in their cortical LifeAct-GFP signal (Figure 4E and Video S7). Next, we used some of the imaging data to create a 3D model of an HEV and followed as single cell transmigrating through the endothelium, entering a TEM pocket, and eventually escaping the HEV. To follow the LifeAct-GFP expression we monitored a single confocal slice through the middle of the cell along the direction of cell movement (Figures 4F and S3). Together these results show how transmigrating lymphocytes dynamically regulate their F-actin organization to facilitate migration through the HEV barriers to enter the LN parenchyma.

Video S7. Visualizing HEV Pocket Dynamics *In Vivo*, Related to Figure 4 This video shows transmigrated LifeAct-GFP lymphocytes (green cells) accumulated outside the HEV (violet) and forms a dynamic HEV pocket over time. The video is a transverse slice view through an HEV segment with high magnification (∼75X) during regular adoptive lymphocyte trafficking in a resting murine inguinal LN. The isolated LifeAct-GFP lymphocytes were injected intravenously into a wild-type mouse immunostained with Alexa Fluor-647-labeled anti-PECAM-1 mAb 390 for HEV visualization. The video shows that HEV pockets are formed by transmigrated lymphocytes and are highly dynamic due to lymphocyte accumulation and exit. Images were captured at 1 frame per 30 s, and the sequence shows a 40-min period. Representative still images of this sequence are shown in Figure 4E.

### Visualizing F-Actin during Lymphocyte Interstitial Migration

**Figure 5:**
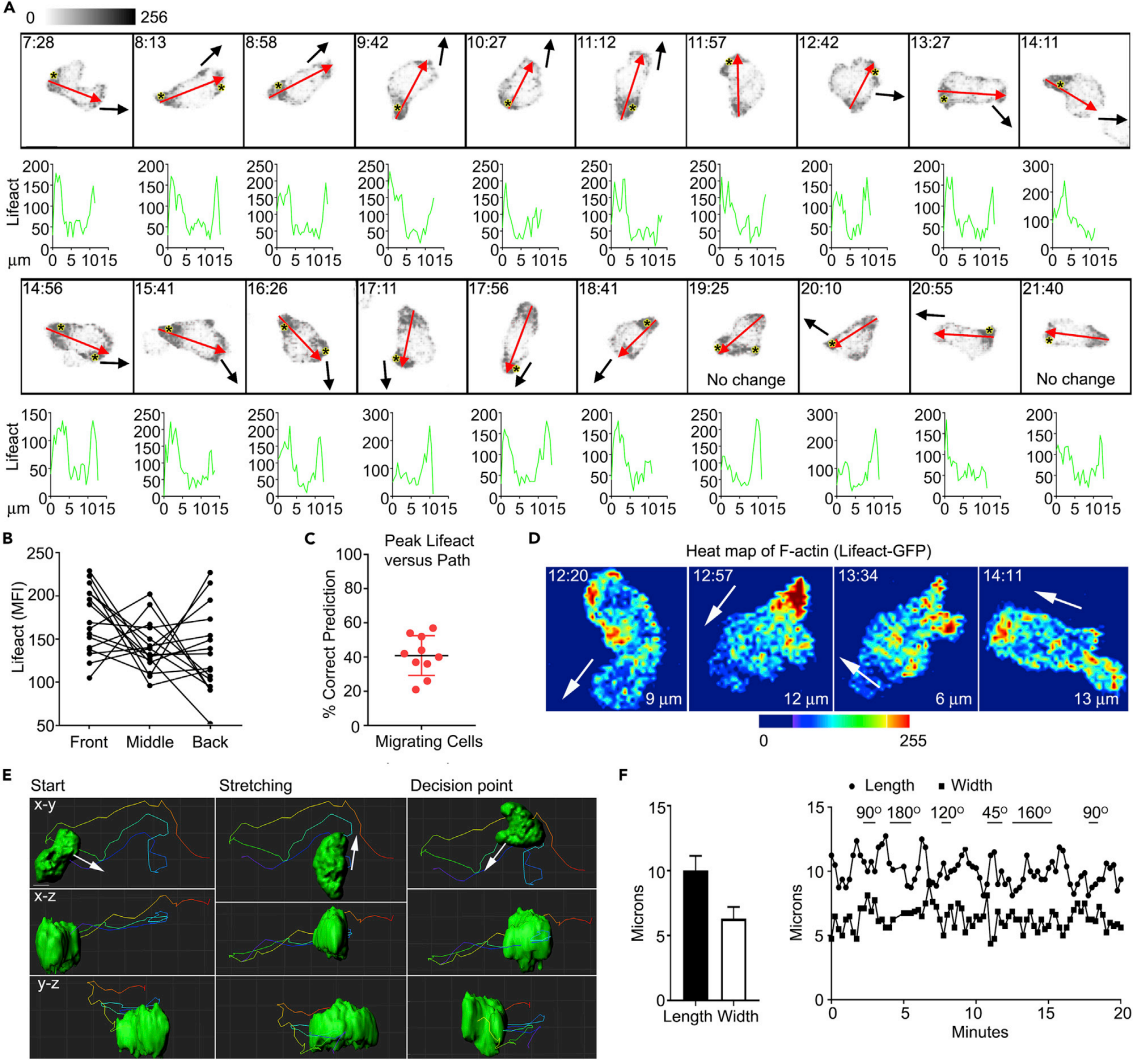
*In Vivo* Imaging of LifeAct-GFP during Lymphocyte Interstitial Migration (A) Representative confocal intravital microscopic images taken from an experiment wherein LifeAct-GFP lymphocytes were adoptively transferred into wild-type host mouse. Image acquired using Ortho Slicer function in Imaris (4.8 μm/5 slices at 0.96 μm). Representative images with indicated times (min:s) are relative to Video S8. Scale bar, 5 μm. Each image represents the tracking of a single cell with the corresponding LifeAct-GFP linear intensity profile below. The red arrow indicates the line where the linear intensity profile was measured and the direction of the original cell migration. The black arrow shows the direction of the cell migration determined from the next time frame. The peak LifeAct signal is shown with an asterisk. Images shown are Z-projections. Data are representative of five experiments with >30 cells observed. (B) Quantitative analysis of LifeAct-GFP distribution at the leading edge, mid-section, and uropod in a migrating cell. A total of 15 cells were analyzed. (C) Utility of peak LifeAct signal in migrating cells to predict subsequent cell movement. A total of 10 cells were tracked every 15 s. Correct prediction occurred if the peak LifeAct signal corresponded (+/− 30°) with the direction of movement. Each dot is the average predictive success of one cell tracked over a minimum of 20 time points. (D) Representative time-lapse images of a migrating LifeAct-GFP cell by heatmap interpretation with indicated times (min:s). White arrows indicate the direction of cell movement. (E) In-depth cell shape analysis and tracking via 3D surface rendering of the LifeAct-GFP signal with X-Y, X-Z, and Y-Z projections shown. The direction of movement is shown with arrows. (F) Evaluating lengths and widths of a migrating cell along the polarity axis (left) and tracking the changes of the migrating cell's lengths and widths over time (right). The changes in cell direction are indicated from 45°–180°.

To examine the behavior of the actin cytoskeleton after lymphocytes exit the HEVs, we examined cells that were freely migrating in the LN parenchyma. Although this represents a complex environment, we hypothesized that the peak intracellular LifeAct GFP signal at the preceding time point in the imaging sequence might help predict the subsequent cell movement. This was not the case as often no correlation existed and frequently the uropod had the highest LifeAct signal. The cell polarity axis did help predict where the cell would move, especially when the cell movement persisted in the same direction. During turns the cell polarity typically lagged that of the path. A single cell shown migrating outside the HEV demonstrates the lack of correlation between peak LifeAct expression and direction of cell movement (Figure 5A, and Video S8). The variable distribution of LifeAct-GFP at the leading edge, in the mid-section, and at the uropod is shown for another migrating cell (Figure 5B). To assess whether imaging at a higher frame rate might reveal a better correlation between the peak Life-Act signal and direction of cell movement we imaged cells every 15 s. However, again the location of the site of peak Life-Act signal in the previous frame poorly predicts the movement of the cell as assessed by its location in the subsequent frame (Figures 5C and S4). Heatmaps of LifeAct-GFP expression at sequential time points show the marked re-organization of F-actin that occurs as a cell migrates within the LN parenchyma (Figure 5D).

Video S8. Visualizing Actin Polymerization during Naive Lymphocyte TEM and Migration *In Vivo*, Related to Figure 5 The video captures lymphocyte actin polymerization at the leading edge with high magnification (∼50X) during regular lymphocyte trafficking in a resting murine inguinal LN. The isolated LifeAct-GFP lymphocytes (green cells) were intravenously injected into a WT mouse immunostained *in vivo* for HEV visualization with Alexa Fluor-647-labeled anti-PECAM-1 mAb 390 (indigo). The video shows a highly motile lymphocyte in the LN interstitium (white arrow) with enhanced actin polymerization (high GFP intensity) polarized at the leading edge of the moving cell. Images were captured at 1 frame per 30 s, and the sequence shows a 10-min period. Still images of this sequence are shown in Figure 5A.

Several recent *in vitro* studies have highlighted the importance of actin-based-protrusions in pseudopod formation and leukocyte path finding in complex 3D environments (Hons et al., 2018, Ridley et al., 2003). Because of the improved resolution of the imaging system we could assess pseudopod formation in relation to path direction *in vivo*. Following their adoptive transfer, we focused on cells that had transmigrated and had subsequently migrated away from the HEVs. We used the surface rendering function in Imaris software to approximate the shape of the cell as it migrates using the LifeAct-GFP signal. The results of this analysis are shown looking in the X-Y, X-Z, and Y-Z projections (Figures 5E and S5, Videos S9 and S10). Usually the migrating cells extended broad-based protrusions in the direction of migration, although occasionally such protrusions could not be identified. However, the cell also extended broad-based pseudopods that did not lead to migration in the direction of the protrusion. During migration the cells typically remain persistently polarized. Plotting the cell length versus the width of the cell along the polarity axis during migration reveals how the cell lengthens and shortens. During turns the cell often rounded up before extending in the direction of the new path (Figure 5F and Video S11). These dynamic cell shape changes likely help drive the cell's motility in the low-adhesive environment found in the LN parenchyma.

Video S9. Visualizing *In Vivo* Lymphocyte Interstitial Migration in x-y Projection, Related to Figure 5 This video shows migration of a LifeAct-GFP lymphocyte in the LN interstitium with high magnification (∼75X). The video is viewed in an X-Y projection after surface-rendering 3D reconstruction with tracking of the cell. Original images were captured at 1 frame per 15 s, and the sequence shows a 20-min period. Still images of this sequence are shown in Figure 5E.

Video S10. Visualizing *In Vivo* Lymphocyte Interstitial Migration in x-z Projection, Related to Figure 5 This video shows the previous migrating LifeAct-GFP lymphocyte in an X-Z projection after surface-rendering 3D reconstruction cell tracking. Original images were captured at 1 frame per 15 s, and the sequence shows a 20-min period. Still images of this sequence are shown in Figure 5E.

Video S11. Visualizing Lymphocyte Reversing Direction of Interstitial Migration, Related to Figure 5 This video shows cell tracking from the top view of a migrating LifeAct-GFP lymphocyte in the LN interstitium with high magnification (∼75X), after surface-rendering 3D reconstruction. Original images were captured at 1 frame per 15 s, and the sequence shows a 10-min period.

### Inhibition of F-Actin Formation Interferes with Lymphocyte-Endothelium Interactions and TEM

As adoptively transferred lymphocytes rapidly access HEVs, we could assess the impact of F-actin inhibitors on lymphocyte adhesion and TEM *in vivo.* We tested four inhibitors at relatively high concentrations and focused our analysis on the half hour after lymphocyte transfer because of the progressive drug washout (Barzilai et al., 2017, Fritz-Laylin et al., 2017, Peng et al., 2011, Rizvi et al., 2009). We chose latrunculin B, which blocks new actin polymerization by sequestering the free actin monomer pool; blebbistatin, which directly inhibits myosin II activity and prevents its functional role in restructuring the F-actin network; SMIFH2, which inhibits formins preventing the generation of unbranched F-actin; and CK-666, which inhibits the Arp2/3 complex inhibiting the nucleation of branched F-actin filaments (Barzilai et al., 2017, Fritz-Laylin et al., 2017, Peng et al., 2011, Rizvi et al., 2009). We first verified *in vitro* that the inhibitors reduced lymphocyte chemotaxis in a standard chemotaxis assay. We chose CCL19 because of its known role in the entrance of lymphocytes into LNs. We checked the response of B cells and CD4 T cells isolated from spleen. Not surprisingly, latrunculin B completely blocked chemotaxis, whereas blebbistatin and CK666 each modestly reduced the percentage of cells migrated into the bottom chamber. The formin inhibitor SMIFH2 proved as efficacious as latrunculin B in inhibiting chemokine-directed migration *in vitro* (Figure S6).

**Figure 6:**
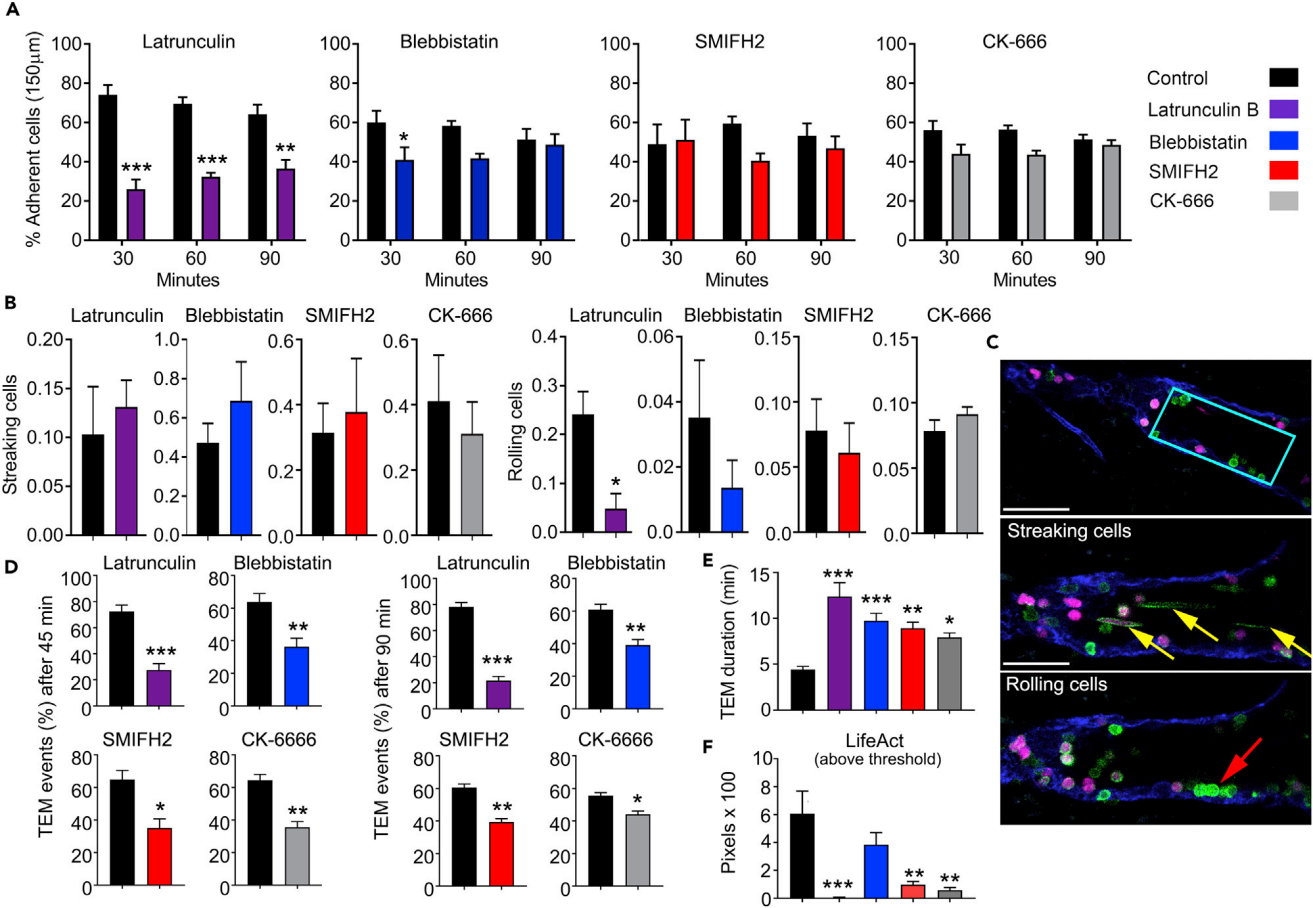
Effects of Inhibitors of Actin Filament Organization on Lymphocyte-EC Interactions and TEM Migration (A) Test of indicated inhibitors and their effects on lymphocyte adherence with length of 150 μm on HEV luminal surfaces. Adherent cells were counted at 30, 60, and 90 min after initial lymphocyte injection into the host mice. Data are representative of three to six experiments per group with one mouse per experiment. More than 100 events were analyzed per group. Statistics: mean ± SEM and unpaired t test performed with Prism comparing drug treated with control. (B) Test of inhibitors on lymphocyte rolling along HEVs by quantifying streaking cells (top row) and rolling cells (bottom row) for 60 min after initial lymphocyte injection. Data are representative of three to six experiments per group with one mouse per experiment. Statistics: mean ± SEM and unpaired t test performed with Prism comparing drug treated with control. (C) Visual representation of streaking and rolling cells by taking an HEV segment of 40 × 15 × 100 μm volume (first panel, cyan box). Yellow arrows indicate streaking cells, and red arrow shows a rolling cell along the HEV (indigo) relative to Video S12. Scale bars, 50 μm and 40 μm. Confocal images shown as Z-projections. (D) Total transmigrated cells were measured at 45 and 90 min following lymphocyte injection as shown in Figure S5A. Statistics: mean ± SEM and unpaired t test performed with Prism comparing drug treated with control. (E) Duration of lymphocyte TEM of control cells versus cells treated with inhibitors. Data are representative of three to six experiments per group with 1 mouse per experiment with >50–105 TEM events analyzed per group in TEM duration measurements. Statistics: mean ± SEM and unpaired t test performed with Prism comparing drug treated with control. (F) Effect of drugs on LifeAct signal intensity during lymphocyte migration on the HEV endothelium. Inhibitor-treated or control cells were imaged every 15–20 s for a minimum of 2 min on the HEV endothelium. Ortho Slicer function in Imaris was used to collect the GFP signal. Individual images were analyzed in Photoshop to determine the number of pixels above a threshold of 125 in the green channel. Statistics: mean ± SEM of unpaired t test performed with Prism comparing data from drug treated and control cells. *p < 0.05, **p < 0.01, ***p < 0.001.

To identify the treated cells *in vivo* we labeled them with CellTracker orange before adoptive transfer. We measured the number of adherent cells at 30, 60, and 90 min following cell injection (Figure 6A). The latrunculin B treatment decreased adhesion at each measured time point. Blebbistatin treatment modestly decreased the number of adherent cells at 30 min, whereas SMIFH2 and CK-666 had no observable effects. Although our image acquisition rate was insufficient to image rolling cells, we could detect cell streaks and cells captured moving along the endothelium in sequential image slices, which we termed rolling cells. None of the inhibitors significantly changed the number of streaking cells (Figure 6B). Latrunculin B reduced the number of rolling cells, whereas the other inhibitors lacked a significant effect (Figure 6B). A visual representation of how we obtained these data is shown (Figure 6C and Video S12).

Video S12. Visualizing Streaking and Rolling Lymphocytes *In Vivo*, Related to Figure 6 This video shows an HEV segment with control versus actin inhibitor (latrunculin B)-treated cells interacting with HEV ECs on the luminal side. The isolated LifeAct-GFP lymphocytes were divided into the following groups: (1) unstained, untreated LifeAct-GFP (green) cells and (2) CellTracker Orange-stained, actin inhibitor-treated LifeAct-GFP (magenta) cells. These cells were then injected into a WT mouse *in vivo* labeled for HEV visualization with Alexa Fluor-647-labeled anti-PECAM-1 mAb 390 (indigo). The video shows streaking and rolling cells within the HEV lumen. Images were captured at 1 frame per 30 s with ∼40X magnification. The sequence shows a 60-min period. Representative still images of this sequence are shown in Figure 6C.

Next, we assessed TEM comparing the untreated to the drug-treated cells. Details of the analysis of lymphocyte TEM and LifeAct expression and representative videos are shown (Figure S7, and Videos S13, S14, S15, and S16). We used the imaging data to assess the impact of the drug treatments on total lymphocyte TEM and on TEM duration. Each of the drugs reduced the number of TEM events and increased the average TEM duration compared with the control cells. Latrunculin B treatment had the most detrimental effects (Figures 6D and 6E). Latrunculin B, SMIFH2, and CK-666-treated cells tended to have a lower LifeAct-GFP footprint than did the control cells, which typically exhibited a dynamic alteration in their LifeAct-GFP signal during migration on the endothelium (Figure 6F).

Video S13. Visualizing the Impact of Latrunculin B on Lymphocyte Trafficking through HEVs, Related to Figure 6 This video shows the result of inhibiting new F-actin formation. Isolated LifeAct-GFP lymphocytes were unstained, untreated LifeAct-GFP (green) cells, or CellTracker Orange-stained, latrunculin B-treated LifeAct-GFP (magenta) cells. Both sets of cells were injected intravenously into a mouse *in vivo* labeled for HEV visualization with Alexa Fluor-647-labeled anti-PECAM-1 mAb 390 (indigo). Images were captured at 1 frame per 30 s with ∼25X magnification. The sequence shows a 60-min period.

Video S14. Visualizing the Impact of Blebbistatin on Lymphocyte Trafficking through HEVs, Related to Figure 6 This video shows the result of inhibiting myosin IIA. Isolated LifeAct-GFP lymphocytes were unstained, untreated LifeAct-GFP (green) cells, or CellTracker Orange-stained, blebbistatin-treated LifeAct-GFP (magenta) cells. These cells were transferred into a mouse *in vivo* labeled for HEV visualization with Alexa Fluor-647-labeled anti-PECAM-1 mAb 390 (indigo). Images were captured at 1 frame per 30 s with ∼25X magnification. The sequence shows a 60-min period.

Video S15. Visualizing the Impact of SMIFH2 on Lymphocyte Trafficking through HEVs, Related to Figure 6 This video shows the result of inhibiting formin mediated new actin nucleation. The isolated LifeAct-GFP lymphocytes were unstained, untreated LifeAct-GFP (green) cells or CellTracker Orange-stained, SMIFH-2-treated LifeAct-GFP (magenta) cells. These cells were then transferred to a mouse *in vivo* labeled with Alexa Fluor-647-labeled anti-PECAM-1 mAb 390 to visualize HEV vasculature (indigo). Images were captured at 1 frame per 30 s with ∼25X magnification. The sequence shows a 60-min period.

Video S16. Visualizing the Impact of CK-666 on Lymphocyte Trafficking through HEVs, Related to Figure 6 This video shows the result of inhibiting actin branch formation. The lymphocytes were unstained, untreated LifeAct-GFP (green) cells or CellTracker Orange-stained, Arp2/3-treated LifeAct-GFP (magenta) cells. These cells were transferred to a mouse *in vivo* labeled for HEV visualization with Alexa Fluor-647-labeled anti-PECAM-1 mAb 390 (indigo). Images were captured at 1 frame per 30 s with ∼25X magnification. The sequence shows a 60-min period.

**Figure 7:**
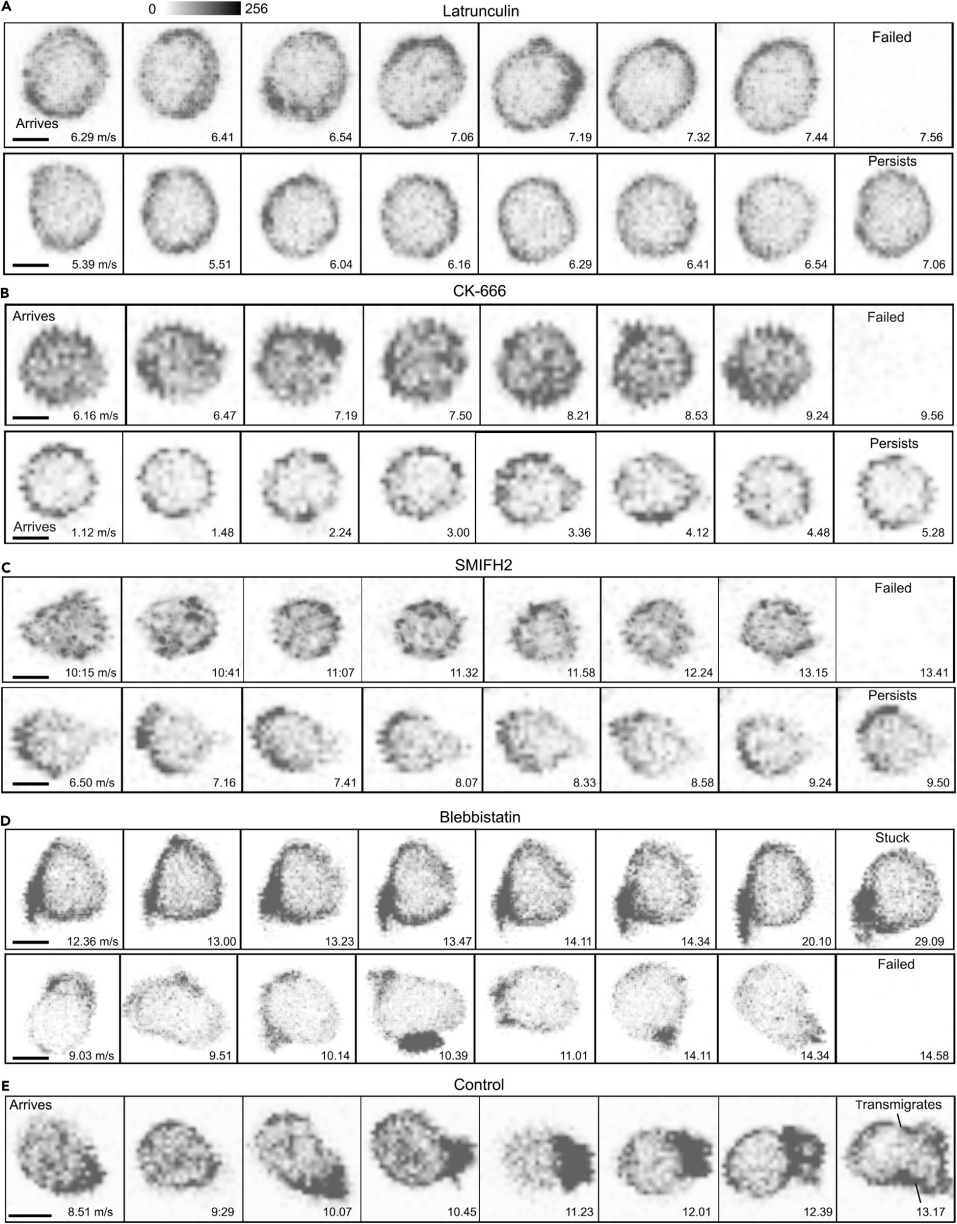
Visualizing the Impact of Inhibiting F-Actin Filament Organization on TEM Test of various inhibitors and their effects on cell polarity and actin filament organization during lymphocyte TEM. Serial images focusing on LifeAct localization in lymphocytes migrating on the HEV endothelium; only the LifeAct signal is shown with indicated times (min:s). Scale bar, 5 μm. Effects of latrunculin B (A), CK-666 (B), SMIFH2 (C), and blebbistatin (D) and control (E) on lymphocyte polarity, mobility, adherence, and TEM. LifeAct-GFP (green) channel and interpreted with gray scale. All confocal images shown are Z-projections from Ortho Slicer function in Imaris (4.8 μm/5 slices at 0.96 μm).

Representative images of individual cells adherent to the HEVs within 30 min of transfer are shown (Figure 7). The adherent latrunculin-treated cells remained unpolarized and often failed to find TEM sites on the endothelium (Figure 7A and Video S13). The CK-666- and SMIFH2-treated cells exhibited reduced motility on the HEVs, difficulty in finding a TEM site, and sometimes failure to remain adherent to the HEV (Figures 7B and 7C, Videos S15 and S16). The blebbistatin-treated cells occasionally adopted unusual morphologies while persistently stuck on the endothelium (Figure 7D and Video S14). These results indicate that inhibiting F-actin formation with latrunculin limits lymphocyte capture and adhesion to HEVs. Interfering with formins, Arp2/3, and with myosin II activity minimally affected lymphocyte adhesion to HEVs, but did impair TEM site localization, and TEM. For comparison purposes the LifeAct-GFP profile of a typical control cell that quickly undergoes TEM is shown (Figure 7E).

## Discussion

Despite significant efforts and progress in the understanding of the mechanisms that regulate the transmigration of lymphocytes through HEV vessel walls, many aspects of this response remain unknown (Girard et al., 2012, Ley et al., 2007). A major limiting factor has been the shortage of advanced imaging techniques for distinct tracking and visualization of lymphocytes around the identified components of the HEV walls. To support our interest in better understanding lymphocyte trafficking and the signaling mechanisms that control it, we have established an imaging system optimized for distinct tracking and analysis of the transmigration of adoptive transferred lymphocytes through HEVs. This technique allowed us to investigate the frequency of paracellular and transcellular lymphocyte transmigration in distinct HEV segments. By utilizing LifeAct-GFP mice we could visualize and assess lymphocyte actin filament organization during the TEM process. While results from early electron microscopy studies experiments were inconclusive (Schoefl, 1972, Yamaguchi and Schoefl, 1983) on determining the routes of lymphocyte transmigration via serial section, recent published *in vitro* studies with live-cell imaging techniques also resulted in disparate results depending on the endothelial cells used and the conditions of these *in vitro* experiments. Although paracellular TEM has been reported as the most used transmigration path (Carman et al., 2007, Gerard et al., 2009, Yang et al., 2005), others have also shown transcellular TEM as a dominant route (Millan et al., 2006, Nieminen et al., 2006). Yet these cultured single cell layers do not accurately reflect the cuboidal HEV endothelial cell morphology and lymphoid environment present *in vivo.* Using intravital microscopy, we precisely characterized numerous lymphocyte transmigration events and found mainly paracellular TEM (∼90%), whereas the transcellular TEM events occurred mostly near the PECAM-1-stained junctions of thinner HEV ECs.

The formation of TEM pores on the post-capillary venules is initiated by leukocyte protrusions, which develop into pseudopodia driven by actin polarization at the cell's leading edge (Carman and Springer, 2008, Nourshargh et al., 2010). Previous *in vitro* experiments have revealed the importance of actin filament organization in regulating leukocyte TEM and migration (Carman and Springer, 2008, Renkawitz and Sixt, 2010). However, these studies primarily visualized actin in artificial systems often in the absence of physiological shear stress. In the present study, we successfully visualized F-actin organization and polarization during naive lymphocyte TEM and migration using LifeAct-GFP lymphocytes *in vivo*. Our results show that both naive lymphocyte TEM and migration depend upon actin-driven cell protrusions and cell membrane remodeling. We observed F-actin localizes in the lymphocyte cell cortex in contact with the HEV endothelium during the adhesion process. Subsequently, this actin cytoskeletal arrangement initiates a change in lymphocyte cell shape and the formation of a leading edge. Our results are in line with those of previous *in vitro* studies showing that leukocytes rely on these leading-edge formations to breach mechanical barriers and sustain migratory movements (Renkawitz and Sixt, 2010). In addition, our system allows for quantitative analysis of F-actin filament organization during the changes in lymphocyte shape and cell membrane remodeling that occurs during TEM.

Our previous imaging data showed that many lymphocytes appear to accumulate below HEV ECs, leading to HEV pocket formation (Park et al., 2012). These HEV pockets are highly dynamic structures that continuously change their size and location largely driven by lymphocyte transmigration. Interestingly, the duration for which lymphocytes reside in these HEV pockets varied tremendously. Furthermore, the irregular distribution of the pockets along the length of the HEVs suggests that specific sections of HEVs are more prone to pocket formation than others. These HEV pockets likely allow the HEVs to operate as a homing control site that flexibly adapts its EC shape and structure to accommodate extravasated lymphocytes (Girard et al., 2012). As these HEV pockets act as the rate-limiting step during lymphocyte homing, further *in vivo* studies on HEV pocket structure and function under pathological conditions and during inflammatory reactions is warranted.

In addressing the mechanisms associated with regulating actin filament organization and their role during lymphocyte adhesion and TEM, we used known inhibitors that interfere with different aspects of F-actin remodeling. Not surprisingly, inhibiting new actin polymerization by treating LifeAct-GFP lymphocytes with latrunculin B significantly reduced lymphocyte adhesion and TEM. Several *in vitro* studies have suggested the importance of myosin II in establishing cell polarity and mediating cell migration (Barzilai et al., 2017, Krummel et al., 2014, Wigton et al., 2016). Myosin IIa is responsible for cross-linking actin filaments and contracts the local actin cytoskeleton through its motor activity to generate mechanical force (Wigton et al., 2016), and previous studies have shown the importance of myosin IIa in regulating T cell trafficking both *in vitro* and *in vivo* (Jacobelli et al., 2010, Soriano et al., 2011). In line with other published *in vitro* studies, our *in vitro* data analyzing chemokine-driven chemotaxis also indicate that myosin IIa plays an important role in facilitating lymphocyte transmigration. By pretreating cells with blebbistatin before their adoptive transfer, we demonstrated that myosin IIa inhibition weakly interferes with naive lymphocyte adherence to HEVs and reduces lymphocyte TEM. The imaging data indicated that F-actin polarization and leading-edge formation *in vivo* are partially myosin IIa dependent. Little is known about the importance of F-actin nucleation, which generates new actin filaments, during lymphocyte TEM. Numerous studies have shown that formins and Arp2/3 complexes are the most prominent molecular machineries catalyzing actin nucleation and leukocyte trafficking *in vitro* (Davidson et al., 2018, Kage et al., 2017, Rotty et al., 2017, Sakata et al., 2007, Swaney and Li, 2016, Thompson et al., 2018). Formins are responsible for *de novo* nucleation and new linear actin filaments, whereas the Arp2/3 complex binds to existing filaments and generates new filaments via branching (Kage et al., 2017, Swaney and Li, 2016). Here we revealed that both formins and Arp2/3 complexes facilitate naive lymphocyte TEM *in vivo*. Resolving the disconnect between the *in vitro* and *in vivo* studies with the formin inhibitor needs additional study. One caveat in interpreting the inhibitor results is the progressive drug washout that occurs following cell transfer into a drug free environment. Previous *in vitro* experiments suggest that a 30- to 60-min washout is needed for recovery from the latrunculin treatment. Although we routinely observed the recovery of the inhibitor-treated cells approximately an hour after transfer, we cannot precisely assess the level of drug inhibitor at the time of imaging.

The movement of lymphocytes in the interstitial tissue is essential for physiological and pathological processes such as immune surveillance, tissue homeostasis, and immunological responses. In the LN parenchyma, lymphocytes face a complex, densely packed microenvironment composed of multiple cell types and extracellular matrices (Girard et al., 2012, Mempel et al., 2004, Park et al., 2012, von Andrian and Mempel, 2003). Most studies on the role of actin during leukocyte migration have relied on *in vitro* methods using 2D platforms or 3D tissue mimetics such as collagen matrices (Friedl et al., 2012, Leithner et al., 2016, Liu et al., 2015). Many of these studies have shown actin polymerization at the cell's leading edge during *in vitro* migration (Fritz-Laylin et al., 2017, Leithner et al., 2016). Although these experimental setups allow for environmental, mechanical, and chemical manipulations, they do not truly represent physiological or pathophysiological conditions. In contrast, we have found that the peak F-actin levels within the lymphocyte correlates poorly with the direction of movement within the LN tissue. Lymphocytes were often observed to have their peak LifeAct intensity at their uropod, suggesting a need for strong contractile force to retract the cell's uropod for proper migration. We based this analysis on imaging cells every 15 s as they move in the interstitium. It is possible that we would have found a better correlation between peak LifeAct signal and the subsequent direction of movement had we imaged at a more frequent interval. We did note that the cell polarity orientation, which is highly dependent upon the F-actin cytoskeleton, did help predict the subsequent direction of cell movement.

In conclusion, we have successfully established an intravital imaging platform that allowed us to visualize the interaction of lymphocytes with HEV ECs with refined details. We described the importance of actomyosin networks during naive lymphocyte TEM and migration *in vivo*. These network structures generate the forces needed for lymphocytes to persistently adhere to the HEV lumen, to breach the HEV ECs, and to migrate toward their respective T or B cell zones. In addition, our approach allowed the visualization of the unique structures of the HEVs in greater detail, thus allowing determination of the route of lymphocyte TEM, which predominately occurred via a paracellular route. The confocal intravital imaging technique established here can be adapted to address many peripheral LN-related biological questions, including the dynamics of cell migration, cell-cell interactions, and changes in HEV morphology that occur during pathophysiological conditions.

### Limitation of the Study

By using a confocal microscope for our *in vivo* imaging, we sacrifice the improved depth of imaging and low phototoxicity of the standard two-photon microscope for the improved resolution necessary for our studies.

## Methods

All methods can be found in the accompanying Transparent Methods supplemental file.

## Supplemental Information

Document S1. Transparent Methods and Figures S1–S7
