# Supplementary material for: In Vivo F-Actin Filament Organization during Lymphocyte Transendothelial and Interstitial Migration Revealed by Intravital Microscopy
Source: iScience. 2019 May 30;16:283–97. doi: 10.1016/j.isci.2019.05.040 (PMC6581778; doi:10.1016/j.isci.2019.05.040)
Supplement: Document S1. Transparent Methods and Figures S1–S7 [file mmc1.pdf]

ISCI, Volume *16*

Supplemental Information

In Vivo F-Actin Filament Organization during Lymphocyte Transendothelial and Interstitial Migration Revealed by Intravital Microscopy

Serena L.S. Yan, Il-Young Hwang, Olena Kamenyeva, and John H. Kehrl

Figure S1

A

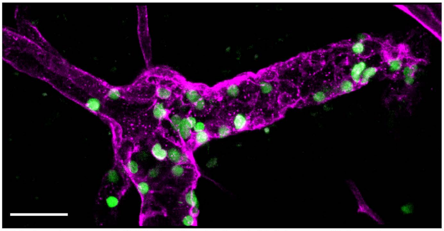

WT + Alexa-647 labelled PECAM1 + cell-tracker labelled cells

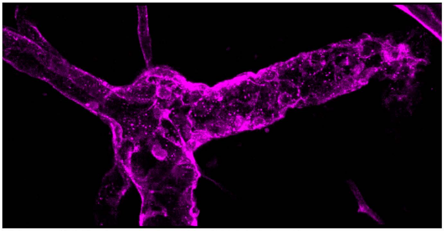

Alexa-647 channel only (PECAM-1)

B

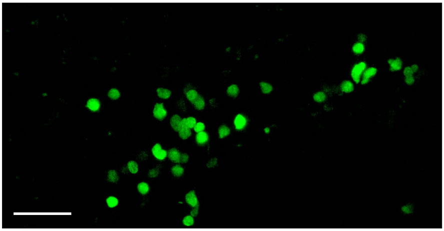

WT + PE labelled control mAb (IgG2a) + cell-tracker labelled cells

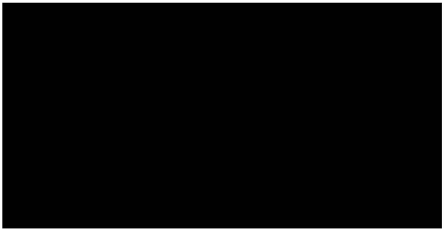

PE channel only

C

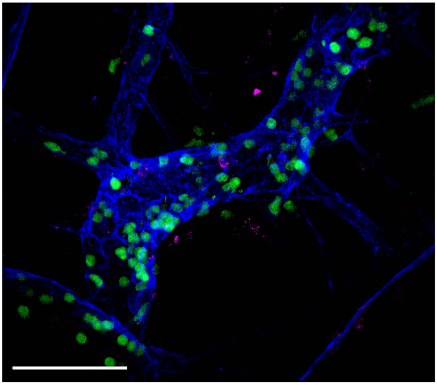

PECAM-1 labelled HEVs (indigo), Gr-1 labelled endogenous neutrophils (magenta), adoptively transferred lymphocytes (green)

D

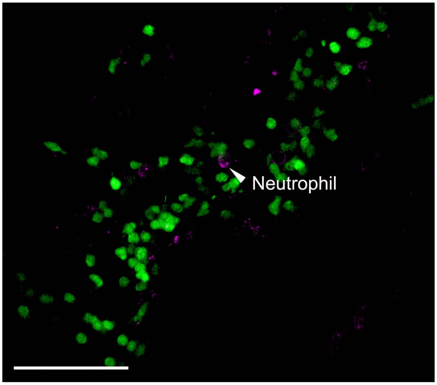

PECAM-1 channel removed

Figure S2

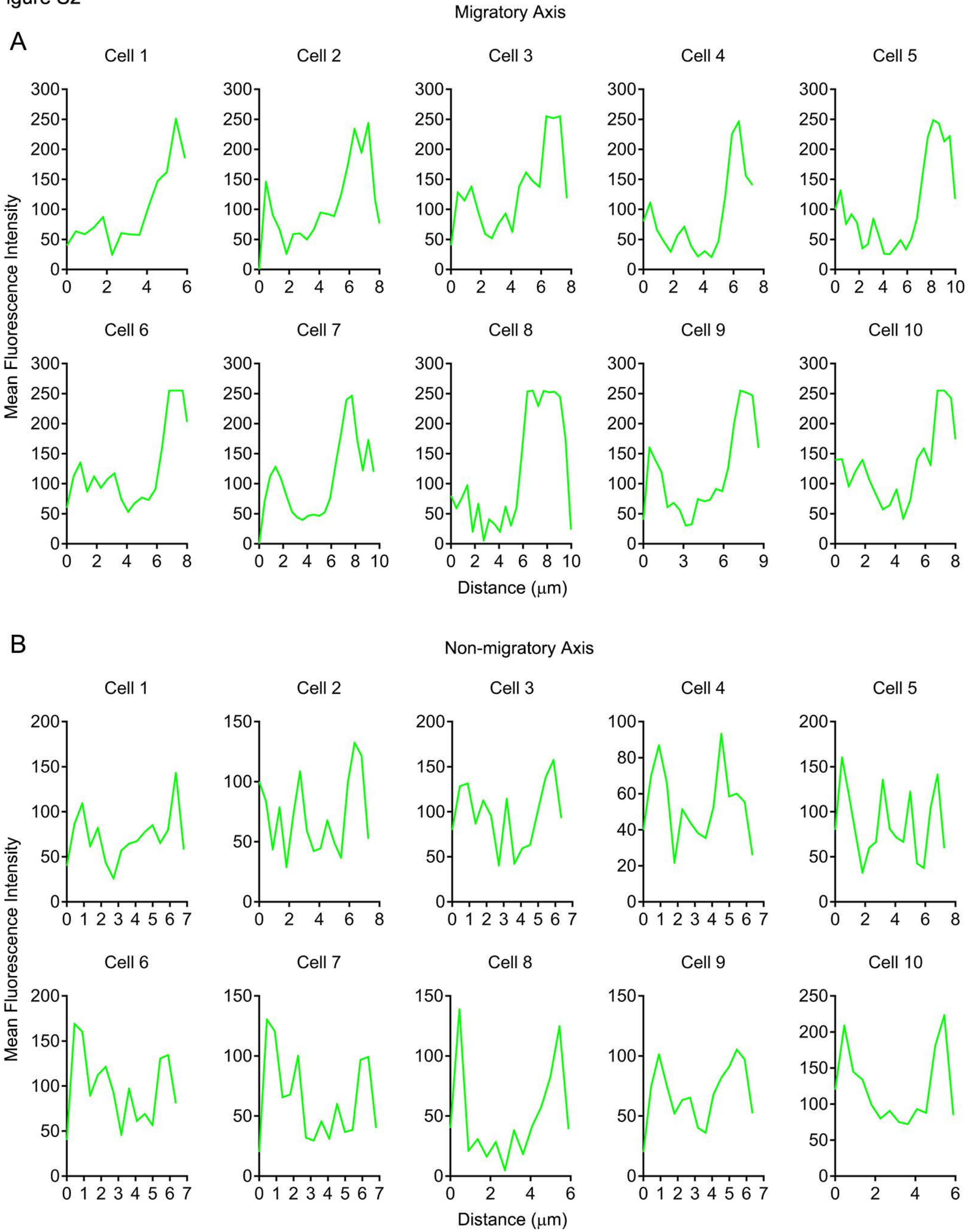
A
Migratory Axis
Cell 1
Cell 2
Cell 3
Cell 4
Cell 5
Cell 6
Cell 7
Cell 8
Cell 9
Cell 10
Mean Fluorescence Intensity
Distance (μm)
B
Non-migratory Axis
Cell 1
Cell 2
Cell 3
Cell 4
Cell 5
Cell 6
Cell 7
Cell 8
Cell 9
Cell 10
Mean Fluorescence Intensity
Distance (μm)

Figure S3

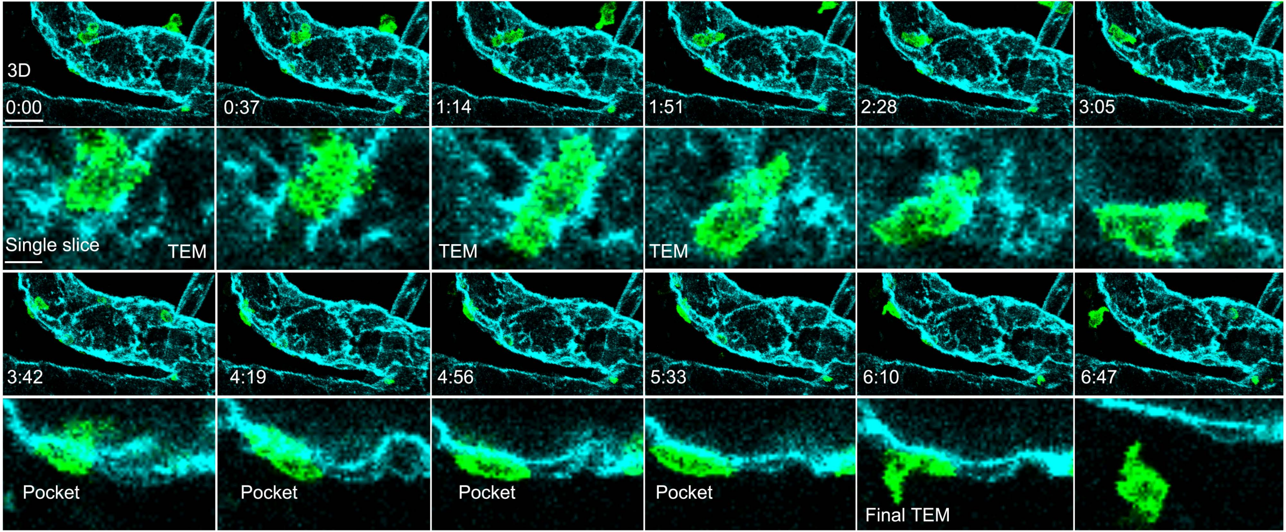
3D
0:00
0:37
1:14
1:51
2:28
3:05
Single slice
TEM
TEM
TEM
3:42
4:19
4:56
5:33
6:10
6:47
Pocket
Pocket
Pocket
Pocket
Final TEM

Figure S4

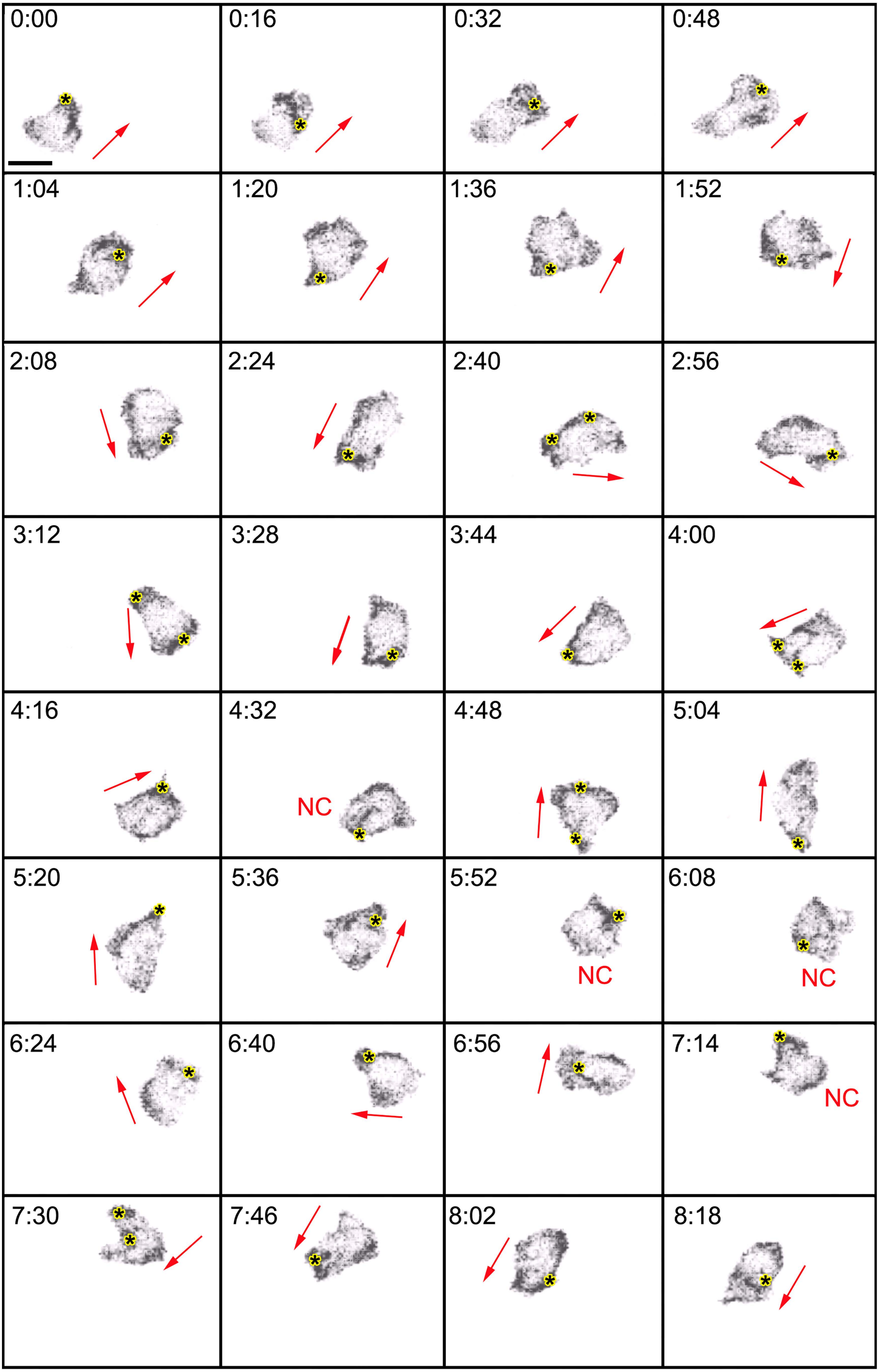
0:00
0:16
0:32
0:48
1:04
1:20
1:36
1:52
2:08
2:24
2:40
2:56
3:12
3:28
3:44
4:00
4:16
4:32
NC
4:48
5:04
5:20
5:36
5:52
NC
6:08
NC
6:24
6:40
6:56
7:14
NC
7:30
7:46
8:02
8:18

Figure S5

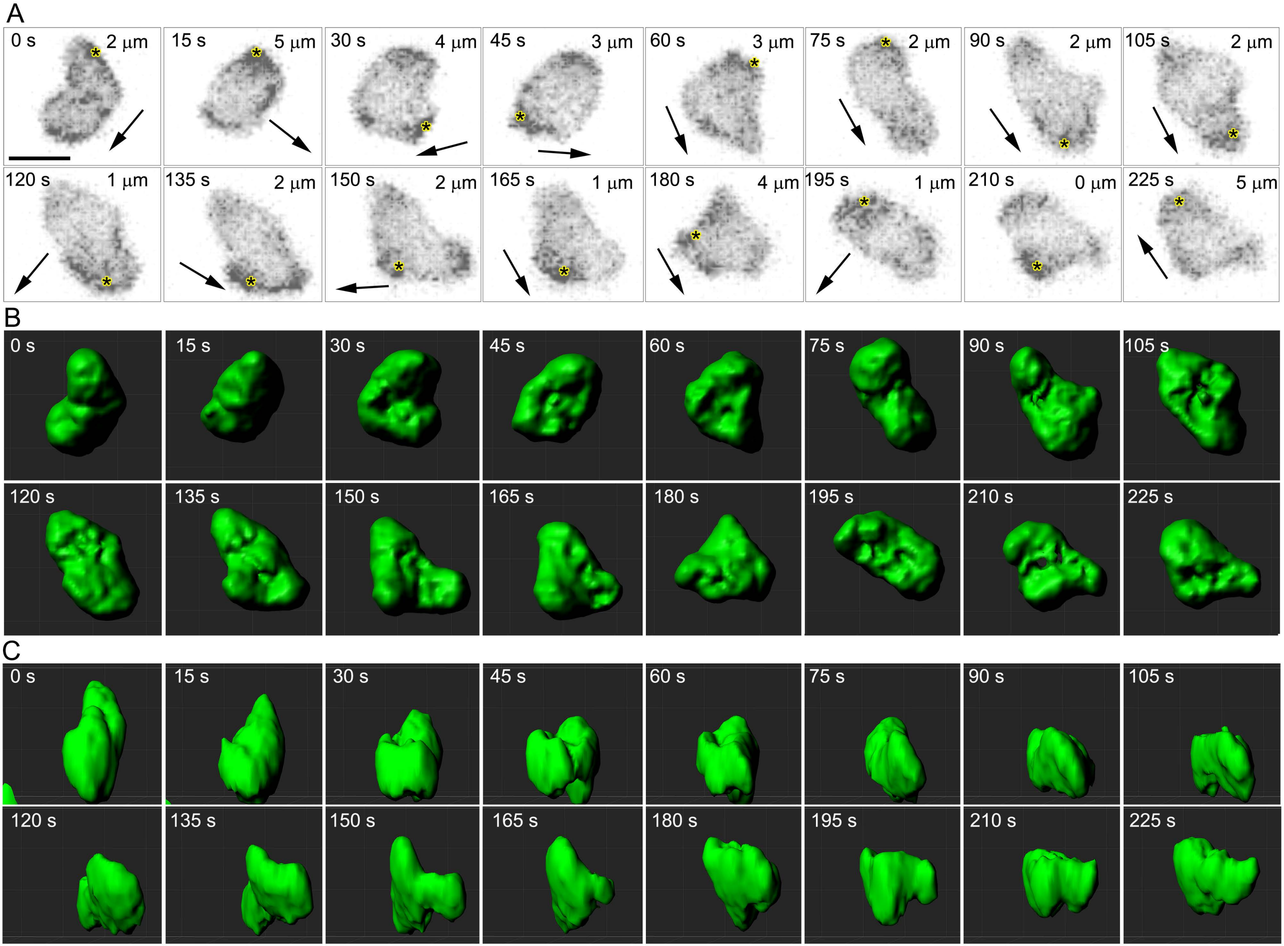
A
0 s
2 µm
15 s
5 µm
30 s
4 µm
45 s
3 µm
60 s
3 µm
75 s
2 µm
90 s
2 µm
105 s
2 µm
120 s
1 µm
135 s
2 µm
150 s
2 µm
165 s
1 µm
180 s
4 µm
195 s
1 µm
210 s
0 µm
225 s
5 µm
B
0 s
15 s
30 s
45 s
60 s
75 s
90 s
105 s
120 s
135 s
150 s
165 s
180 s
195 s
210 s
225 s
C
0 s
15 s
30 s
45 s
60 s
75 s
90 s
105 s
120 s
135 s
150 s
165 s
180 s
195 s
210 s
225 s

Figure S6

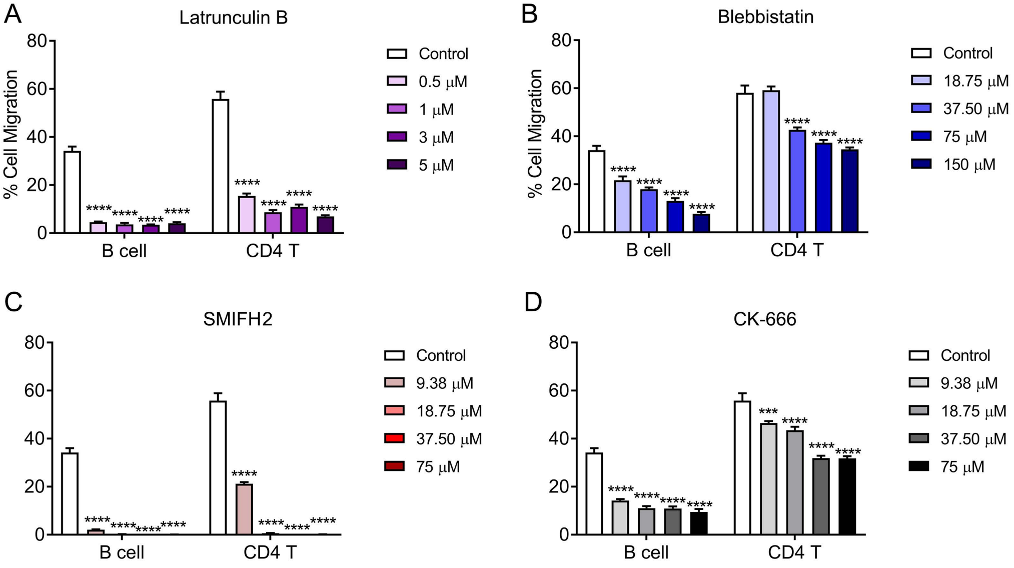

A
Latrunculin B
% Cell Migration
80
60
40
20
0
B cell
CD4 T
****
Control
0.5 μM
1 μM
3 μM
5 μM
B
Blebbistatin
% Cell Migration
B cell
CD4 T
Control
18.75 μM
37.50 μM
75 μM
150 μM
C
SMIFH2
B cell
CD4 T
Control
9.38 μM
18.75 μM
37.50 μM
75 μM
D
CK-666
B cell
CD4 T
***
Control
9.38 μM
18.75 μM
37.50 μM
75 μM

Figure S7

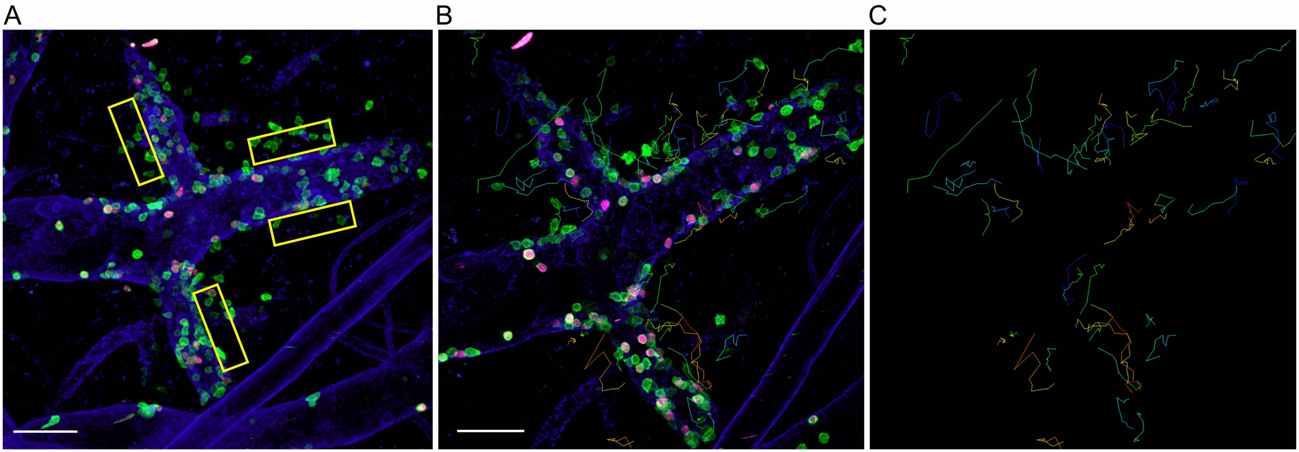

Supplemental Figure Legends

Figure S1. *In vivo* labeling HEVs with PECAM-1 and Gr-1 antibodies, Related to Figure 2 and 3.

(A) i.v. injection of Alexa Fluor-647-anti-PECAM-1 mAb (PECAM-647, clone 390, 25 µg in 100 µL saline) resulted with strong and specific labeling of HEVs (right, magenta) in the mouse inguinal LN. The i.v. injected CellTracker-labeled lymphocytes (green) isolated from WT donor mouse can be clearly identified as intraluminal or extraluminal.

(B) PE-conjugated IgG2a isotype control Ab injected i.v. into a WT mouse does not label the HEV vasculature. (A,B) Scale bars = 30 µm.

(C) WT host with PECAM-1-labeled HEVs (indigo), Gr1-labeled neutrophils (magenta) and adoptive transferred CellTracker-labeled lymphocytes (green) isolated from WT donor mice. (D) By focusing on Alexa-555 (neutrophils) and GFP (lymphocytes) channels, only very few neutrophils appear inside the HEV lumen showing no host neutrophil TEM at HEVs under resting conditions. Scale bar = 50 µm. All representative confocal images shown are Z-projections.

Figure S2. LifeAct-GFP localization as cell approaches a TEM site on the HEV endothelium, Related to Figure 4.

(A-B) Representative LifeAct-GFP intensity profiles of adoptive transferred lymphocytes during TEM through HEVs used to evaluate F-actin distribution within the transmigrating cells along the (A) migratory axis and (B) non-migratory axis. (A) MFI (green) of 10 LifeAct-GFP cells undergoing TEM were measured along their migratory axis with distance (µm) corresponding to uropod (tail) – leading edge direction. (B) MFI of the same 10 LifeAct-GFP cells in (A) were measured again along their non-migratory axis, which was perpendicular to the migratory axis.

Figure S3. *In vivo* imaging of LifeAct-GFP lymphocyte around HEV during TEM, Related to Figure 4.

Time-lapse images following a LifeAct-GFP (green) undergoing initial TEM by breaching the HEV (cyan) barrier, then becomes flattened and migrate between the HEV and basement membrane interface (indicated times, mm:ss). Top rows show 3D reconstruction (Scale bar = 20 µm) while bottom rows (Scale bar = 5 µm) reveal correlating transverse sections through the middle of the cell. Data are representative of 5 experiments with >30 events observed. All representative confocal images shown are Z-projections.

Figure S4. Peak F-actin localization during interstitial lymphocyte migration, Related to Figure 5.

Adoptively transferred LifeAct-GFP lymphocyte migrating within the lymph node parenchyma after exiting an HEV with indicated times (minutes/seconds). Images stacks collected approximately every 15 seconds and images acquired using Orthoslicer function in Imaris and displayed as Z-stack images (5.8 µm/0.96 µm slice). Data shown in grey scale to visualize F-actin distribution. Peak Life-Act localization indicated by a yellowed asterisk. Direction of cell movement shown with a red arrow. The cell remained within the imaging field shown. Scale bar = 6 µm.

Figure S5. Assessing changes in cell shape and the distribution of F-actin during lymphocyte interstitial migration, Related to Figure 5.

(A-C) Representative serial images of an adoptively transferred LifeAct-GFP lymphocyte migrating in the LN interstitium with indicated times (seconds). Scale bar = 6 µm. (A) A LifeAct-GFP lymphocyte (green channel only) from the top (X-Y view). Data shown in grey scale to visualize F-actin distribution. The black arrows indicate the direction of the cell movement and the distance of the cell movements are shown on top right of each image. Cell shape analysis in X-Y view (B) and X-Z view (C) after 3D surface rendering using LifeAct-GFP signal. Data shown in grey scale to visualize F-actin distribution. Peak Life-Act localization indicated by a yellowed asterisk.

Figure S6. Effect of inhibiting F-actin dynamics on lymphocyte chemotaxis *in vitro*, Related to Figure 6.

Cell migration in chemotaxis assays used to evaluate the responses of splenic B and CD4 T cells to CCL19 (100ng/mL). Splenic lymphocytes prepared from WT mice treated with Latrunculin B (A), Blebbistatin (B), SMIFH-2 (C), and CK-666 (D) from low to high doses were subjected to chemotaxis through uncoated 5 µm-pore filters. Results show % of input that responded to CCL19 in the lower chamber; data averaged from n = 6. Statistical differences were calculated by two-way ANOVA. Error bars = $\pm$ SEM. $^{***}P<0.001$, $^{****}P<0.0001$ for cells exposed to CCL19 media to cells in control media. Experiment repeated 3 times with similar results.

Figure S7. Data analysis of total lymphocyte TEM and migration, Related to Figure 6.

(A) A representative image of a WT mouse with PECAM-1 labeled HEVs (indigo), adoptive transferred LifeAct-GFP lymphocytes either untreated (green) or treated with Latrunculin B (magenta). At t = 90 min, yellow quadrants (50 µm x 100 µm) indicate areas selected for total TEM analysis. Scale bar = 50 µm. For each mouse analyzed, 3-5 quadrants are drawn for total TEM quantification.

(B,C) After TEM, cells can be identified and tracked for 10 min outside HEVs to measure their displacement (µm) and speed (µm/s) as shown by the track lines in this representative image during analysis. Scale bar = 50 µm. All representative confocal images shown are Z-projections.

Transparent Methods

Mice. All mice were maintained in specific-pathogen-free conditions at an Association for Assessment and Accreditation of Laboratory Animal Care-accredited animal facility at the NIAID. All animal experiments and surgical procedures were performed under a study protocol approved by NIAID animal Care and Use Committee (National Institutes of Health).

Confocal intravital microscopy imaging of mouse inguinal LN. LifeAct-GFP bone marrow chimeric mice and adoptive transfer of lymphocytes isolated from donor LifeAct-GFP or C57BL/6 (WT) then labeled with 0.5 µM CellTracker Orange CMTMR dye for i.v. injection into C57BL/6 host mice. EC junctions and HEV vasculature were labeled with Alexa Fluor 555 or 647-labeled mAb 390 to PECAM-1 (25 µg injected

i.v.). For intravital microscopy, anaesthesia of mice was initiated with i.p. injection of Avertin (300 mg/kg) and the inguinal LN was exposed by surgically removing surrounding tissue carefully without perturbing blood and lymph vessels. After surgery, the mice were transferred onto an imaging stage and anaesthetized using isoflurane (Baxter; 2% for induction, 1 – 1.5% for maintenance, vaporized in an 80:20 mixture of oxygen and air), then placed into a temperature control chamber on the Leica SP8 inverted 5 channel confocal microscope equipped with a motorized stage and 4 hybrid ultra-sensitive detectors (Leica Microsystems), and a temperature-controlled environmental chamber (NIH Division of Scientific Equipment and Instrumentation Services).

HEVs of IV – V order with 20 – 40 µm in diameter were selected for analysis of lymphocyte – vessel wall interactions. *Z*-stacks of images were captured by confocal microscopy with a single-beam Leica TCS-SP8 confocal laser-scanning microscope equipped with argon and helium neon lasers and incorporating a 25x water-dipping objective (numerical aperature, 1.0; Leica). This microscope incorporates optical zoom function, and most image sequences were captured at a final magnification of ~x40-x60. Images were acquired by sequential scanning of the 488-nm channels for LifeAct GFP lymphocytes, 561-nm for CMTMR-labeled lymphocytes or AlexaFluor 555-antibody to PECAM-1, and 633-nm channels for AlexaFluor 647-antibody to PECAM-1 at a resolution of 1024 x 1024 pixels, which corresponds to a voxel size of approximately 0.25 x 0.25 x 0.7 µm in the *x* – *y* – *z* planes. Stacks of images of optical sections ~ 1 µm in thickness were routinely acquired at intervals 15 – 60 sec, and with the incorporated resonance scanner of 8,000 Hz. This method allowed acquisition of full three-dimensional confocal images of HEVs, which yielded high-resolution four-dimensional videos (real-time in three dimensions) of dynamic events. Tissues were firmly maintained for all preparations without movement interfering with blood flow or image acquisition. After acquisition, sequences of *z*-stack images were analyzed with Imaris four-dimensional modeling (Bitplane) for Huygens Professional software.

Sequences of *Z*-stack images were analyzed with Imaris, which renders stacks of optical sections into three-dimensional models, allowing analysis of the dynamics of lymphocyte – EC interactions. As the work here was aimed at developing a system with high resolution and better color-separation to visualize the extent of lymphocyte actin polymerization and route of TEM across the HEV. With this approach, the development of a confocal intravital imaging technique allowed this study with high clarity and considerable temporal and spatial resolution. Huygens Professional software was used for analysis of intensity profiles of LifeAct-GFP or AlexaFluor-555-mAb to PECAM-1 (390).

Image analysis of lymphocyte activity. Confocal intravital microscopy images were analyzed for profile and dynamics of lymphocyte TEM. In every video, only TEM events imaged in full in terms of their duration as the cell traversed the HEV and clearly visible in terms of their location and dynamics were used for analysis. Consistently, ~60 – 100 TEM events from a minimum of four mice were analyzed per treatment. The study here was to visualize lymphocyte TEM with focuses on TEM routes (paracellular vs transcellular) and actin polymerization. The imaging settings (described above) were optimal for detailed studies of lymphocyte TEM events from which the following parameters were quantified. First, paracellular TEM

events were identified by the observed EC junctional disruption on the HEVs (labeled with mAb to PECAM-1) and forming a transient pore. Second, transcellular TEM events were identified as those associated with the transient occurrence of pores within the EC body (faintly stained with antibody to PECAM-1) without disruption of EC junctions. Third, the duration of TEM events was calculated as the time between the first frame in which breakage of EC or pore formation of cell body with antibody to PECAM-1 could be seen to the frame where the tracked lymphocyte had fully crossed the EC barrier. Fourth, actin polymerization at the leading edge of the tracked lymphocyte has highest LifeAct-GFP intensity just before TEM. Fifth, mean LifeAct-GFP intensity of lymphocyte surface at initial adhesion measured and compared to different inhibitor treatments.

Mice and bone marrow reconstitutions. Male (20-25g) mice at 5 – 8 weeks of age were used. The principal strain used was that of LifeAct-GFP mice, which enables visualization of F-actin in cells and tissues without significantly interfering with actin dynamics *in vivo* or *in vitro.* This allowed live imaging of actin cytoskeleton and therefore the study of many fundamental biological processes during lymphocyte TEM and migration. Wild type (WT) C57BL/6 mice were purchased from The Jackson Laboratory. All mice were housed under specific pathogen-free conditions. For bone marrow reconstitution, 6 wk-old C57BL/6 (CD45.1) mice were irradiated twice with 550 rad for total of 1100 rad and received bone marrow from LifeAct-GFP mice (CD45.2). The engraftment was monitored by sampling the blood 28 d later. The mice were used 6 – 7 wk after reconstitution. All mice used in this study were 6 – 12 wk of age.

Lymphocyte isolation, preparation and T lymphocyte purification. Splenic lymphocytes were isolated by teasing apart the donor mouse spleen in PBS (Life Technologies), passing through a sterile cell strainer with a 40 µm nylon mash (Fisherbrand) and washed 2 times with PBS before further processing. For experiments using total lymphocytes with actin inhibitors, cells were treated with either 5 µM Latrunculin B, 150 µM Blebbistatin, 75 µM SMIFH-2, or 75 µM CK-666 (All Sigma-Aldrich) for 60 min at room temperature and labeled with 0.5 µM CellTracker Orange CMTMR (Molecular Probes) for 15 min at 37°C, then wash 2 times and resuspend in PBS for *in vivo* injection. For intravital imaging of total lymphocytes, ~ 40 - 50 million cells were injected into 1 host mouse.

Surgery preparation for confocal intravital imaging. WT host mice anaesthetized with i.p. injection of Avertin (300mg/kg, tribromoethanol; Sigma-Aldrich) were stabilized on a surgery board with femoral vein exposed for i.v. injection of AlexaFluor-labeled mAb to PECAM-1, LifeAct-GFP and fluorescently labeled cells using U-100 insulin syringe with 30G needle (BD) under the dissecting microscope. The needle wound was sealed with a cautery pen (Bovie). The abdominal area hair was removed with electric trimmer (Wahl), a midline abdominal incision was made, and the skin gently retracted away from the flesh. The inguinal LN was exposed LN by carefully removing the thin layer of connective tissue covering the area. Once the LN and HEV vessel segments were exposed under a dissecting microscope, the mouse was gently flipped up-side down and secured to the imaging stage. The mouse was transferred on to a temperature-controlled chamber set at 37°C on the Leica SP8 inverted 5 channel confocal microscope equipped with a motorized stage and 2 HyD Ultra-sensitive detectors (Leica Microsystems). The mouse was anaesthetized using

isoflurance (Baxter; 1 – 1.5%, vaporized in an 80:20 mixture of oxygen and air) for the remainder of the intravital imaging experiment.

Flow cytometry and antibodies. Single cells were resuspended in PBS, 2% FBS, and stained with fluorochrome-conjugated or biotinylated antibodies against B220 (RA3-6B2), IgD (11-26c-2a), IgM (R6-60.2), CD24 (M1/69), CD5 (53-7.3), CD4 (GK1.5), CD8 (53-6.7), CD11c (HL3), CD11b (M1/70), CD19 (1D3), CD93 (AA4.1), CD21/35 (4E3), CD23 (B3B4), and CD43 (S7) (all from Biolegend, BD Pharmingen, Thermo Fisher Scientific or R&D Systems). Biotin-labeled antibodies were visualized with fluorochrome-conjugated streptavidin (Thermo Fisher Scientific). LIVE/DEAD® Fixable Aqua Dead Cell Stain Kit (Thermo Fisher Scientific) was used in all experiments to exclude dead cells. Compensation was performed using CompBeads (BD Biosciences) and ArC™ Amine Reactive Compensation Bead individually stained with each fluorochrome. Compensation matrices were calculated with FACSdiva software. Data acquisitions were done on FACSCanto II (BD) flow cytometer and analyzed with FlowJo software version 9 (Treestar).

Chemotaxis assays. Chemotaxis assays were performed using a Transwell chamber (Costar), as previously described (Hwang et al., 2018). Untreated or inhibitor-treated splenic lymphocytes were immunostained for T cell subsets with fluorochrome-conjugated Abs against CD4 and CD8 (eBioscience) washed twice. The cells were then treated with either Latrunculin B (0.5, 1, 3, 5 µM), Blebbistatin (18.75, 37.50, 75, 150 µM), SMIFH-2 (9.38, 18.75, 37.50, 75 µM), or CK-666 (9.38, 18.75, 37.50, 75 µM) (All Sigma-Aldrich) for 60 min at room temperature. The cells were then washed twice, resuspended in complete RPMI 1640 medium and added in a volume of 100 µl to the upper wells of a 24-well Transwell plate with a 3-µm insert. Lower wells contained CCL19 (R&D Systems) at 10 ng/ml and 100 ng/ml in 600 µl complete RPMI 1640 medium. The numbers of cells that migrated to the lower well after 3-h incubation were counted using a MACSQuant flow cytometer (Miltenyi Biotec). The percentage of migration was calculated by the numbers of cells of a given subset that migrated into the bottom chamber divided by the total number of cells of that subset in the starting cell suspension, then multiplying the results by 100. The data were analyzed and calculated using FlowJo software.

Statistics. In vivo results represent samples from four to six mice per experimental group. Results represent mean values of at least triplicate samples for ex vivo experiments. SEM and p values were calculated with t test or ANOVA using GraphPad Prism (GraphPad Software).

Supplementary References

Hwang, I.Y., Boularan, C., Harrison, K., and Kehrl, J.H. (2018). Galphai Signaling Promotes Marginal Zone B Cell Development by Enabling Transitional B Cell ADAM10 Expression. Front Immunol *9*, 687.
